# Dental Calculus Reveals Unique Insights into Food Items, Cooking and Plant Processing in Prehistoric Central Sudan

**DOI:** 10.1371/journal.pone.0100808

**Published:** 2014-07-16

**Authors:** Stephen Buckley, Donatella Usai, Tina Jakob, Anita Radini, Karen Hardy

**Affiliations:** 1 BioArCh, University of York, York, United Kingdom; 2 Istituto Italiano per l'Africa e l'Oriente, Roma; Centro Studi Sudanese e Sub-Sahariani, Treviso, Italy; 3 Department of Archaeology, Durham University, Durham, United Kingdom; 4 University of Leicester Archaeological Services (ULAS), School of Archaeology and Ancient History, University of Leicester, Leicester, United Kingdom; 5 ICREA (Catalan Institution for Research and Advanced Studies), Departament de Prehistòria, Universitat Autònoma de Barcelona, Bellaterra, Spain; Ohio State University, United States of America

## Abstract

Accessing information on plant consumption before the adoption of agriculture is challenging. However, there is growing evidence for use of locally available wild plants from an increasing number of pre-agrarian sites, suggesting broad ecological knowledge. The extraction of chemical compounds and microfossils from dental calculus removed from ancient teeth offers an entirely new perspective on dietary reconstruction, as it provides empirical results on material that is already in the mouth. Here we present a suite of results from the multi-period Central Sudanese site of Al Khiday. We demonstrate the ingestion in both pre-agricultural and agricultural periods of *Cyperus rotundus* tubers. This plant is a good source of carbohydrates and has many useful medicinal and aromatic qualities, though today it is considered to be the world's most costly weed. Its ability to inhibit *Streptococcus mutans* may have contributed to the unexpectedly low level of caries found in the agricultural population. Other evidence extracted from the dental calculus includes smoke inhalation, dry (roasting) and wet (heating in water) cooking, a second plant possibly from the Triticaceae tribe and plant fibres suggestive of raw material preparation through chewing.

## Background

The identification of chemical compounds and identifiable microfossils from dental calculus extracted from archaeological skeletons is providing new insights into dietary composition and biographical detail. These new insights are proving to be useful in accessing evidence for ingested plants, particularly in pre-agrarian periods for which evidence of plant use is rare. Stable isotope analysis has been used extensively to investigate pre-agrarian dietary composition [1] [2]. It provides non-specific identification, principally of primary protein sources, but offers little information on dietary plant sources. Carbon isotope analyses differentiate between C3 and C4 plants, but provide little insight into what the actual plants were. The extraction of chemical compounds and microfossils from dental calculus offers an entirely new perspective on dietary reconstruction. Because of its location within the mouth, dental calculus offers a direct link to material that was inhaled or ingested and its value as a source of biographical information for past human populations has recently become evident in terms of microfossils [3] [4] [5] [6], chemical compounds [7], and as a source of bacterial DNA [8]. Here we offer the results of a combined analytical and morphological analysis of the material extracted from samples of dental calculus from the multi period site of Al Khiday, Sudan. This has enabled us to identify specific food items, inhaled micro-environmental data and the use of teeth for processing plant-based raw materials. The material from Al Khiday is of particular interest as it is a multi-period cemetery. This permits a long-term perspective on the material recovered. Indeed, one of the original aims of this study was to evaluate the limits of survival of both chemical compounds and microfossils given the extreme climate of the Sahara; however, no difference in survival or degradation of materials was encountered through the sequence.

Dental calculus occurs when plaque biofilms accumulate and mineralize. It is associated with chronically poor oral hygiene and is common on archaeological skeletons of all periods. Dental calculus is found around the teeth in the supragingival and subgingival areas and is linked to high levels of carbohydrate consumption due to the sugars that are eventually converted into glucose. Subgingival calculus, which has been identified on material several million years old [9], occurs below the gum-line in the gingival crevice. Subgingival calculus is particularly useful for analysis as it can accumulate and endure indefinitely if it is not mechanically removed [10]. Microbial communities in subgingival calculus are proteolytic rather than sacchrolytic. The metabolic by-products of proteolytic metabolism, such as ammonia, result in localized raised pH. This in turn encourages plaque mineralization as precipitation of calcium phosphate is favoured.

**Figure 1:**
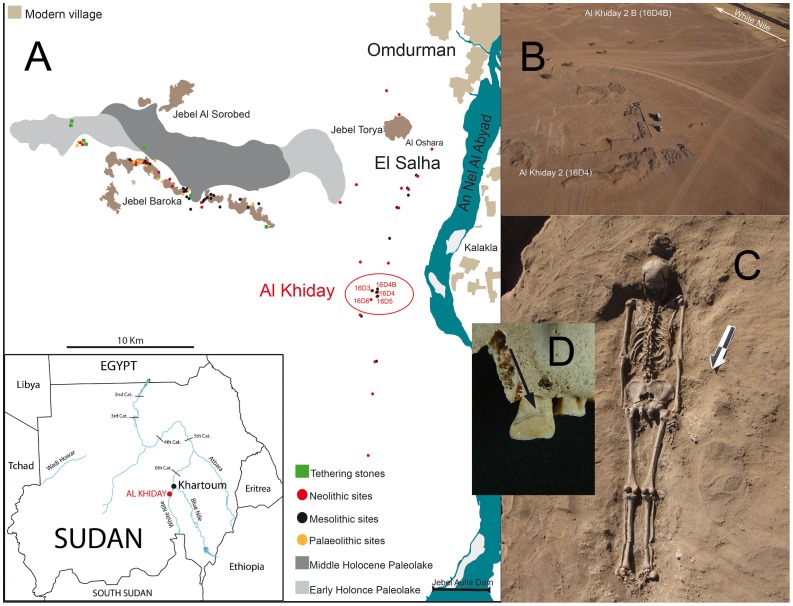
Al Khiday. (A) location map, (b) aerial photograph, excavation, (c) skeleton *in situ*, (d) dental calculus.

Al Khiday is a complex of five archaeological sites which lie 25 kilometres south of Omdurman, on the White Nile, in Central Sudan. Al Khiday 2 is predominantly a burial ground of pre-Mesolithic, Neolithic and Late Meroitic age though it was also used as an occupation site during the Mesolithic period (Figure 1) [11]. The Mesolithic phase is represented by 104 pits which include fireplaces and disposal areas containing Mesolithic material. Although the pre-Mesolithic human remains cannot be directly dated due to insufficient collagen and bio-apatite which have been replaced by environmental carbonatic formations, their graves are cut by the creation of these pits during the Mesolithic which provide a *Terminus ante quem* of 6700 cal. BC [11]. The Neolithic and Meroitic skeletons were dated using charcoal and shells found in the graves [11]. The period covered by these samples stretches from the pre-agricultural fisher-hunter-gatherer based economy through the early Neolithic with its incipient agriculture, and on to the fully developed agricultural context of the Meroitic.

Despite the lack of collagen and low survival of bio-apatite, sufficient carbon and oxygen was retained to conduct some stable isotope analysis on individuals from all periods [11]. The results of these analyses, together with palaeo-environmental studies, have identified a high rainfall environment during the pre-Mesolithic and Mesolithic periods, with increasingly dry climatic conditions during the Neolithic and Meroitic. An ancient wetland area has been identified close by, and Mesolithic faunal remains indicate a relatively lush savannah environment. The δ^13^C isotope analyses of carbonate apatite suggest that human diet in the pre-Mesolithic period was primarily dependent on C_4_-based resources, with a mixture of C_3_- and C_4_-based resources in later periods [11] [12].

## Materials and Methods

Annual excavation and export permits are provided by the National Corporation for Antiquities and Museums (NCAM), Republic of The Sudan, Ministry of Tourism, Antiquities and Wildlife. The project has annual permits since it began in 2005. These are held by the project director (Donatella Usai) and are available if required. Each skeleton is given a unique number and all material from this skeleton is known by this number. This can be seen in Table 1. All material is housed in the Department of Archaeology, Durham University, South Road, Durham DH1 3LE, UK.

**Table 1:** Results.

Sample number	Tooth sampled	Calculus sample	Age	Sex	Period	Starch granules (Sg) and other material (present/absent – P/A)	TD/Py-GCMS	*C. rotundus*
11	Lower right M_3_	Distal supragingival	Adult	Female	Pre-Mesolithic	(Sg) Six type 1. Undamaged (P)		
12	Lower right M_3_	Lingual supragingival	36–45	Male	Pre-Mesolithic	(Sg) Seven, type 1, 2. Undamaged (P)		
35	Upper right M^2^	Buccal supragingival	35+	Male	Pre-Mesolithic	(Sg) One type 1. Undamaged	X	X
41	Lower left M_3_	Distal supragingival	18–35	Female	Pre-Mesolithic	(P)	X	X
55	Lower right M_3_	Buccal + Distal supragingival	18–35	Male	Pre-Mesolithic	(Sg) One type 2. Undamaged		
64	Upper right M^3^	Buccal supragingival	18–35	Male	Pre-Mesolithic	(Sg) One type 1. Undamaged (P)	X	X
77	Lower left M_3_	Lingual supragingival	35+	Female	Pre-Mesolithic	(Sg) One type 2. Undamaged (P)	X	
88	Upper left M^1^	Buccal supragingival	18–35	Male	Pre-Mesolithic	(Sg) One type 1. Undamaged.		
111	Lower right M_1_	Buccal supragingival	Adult	Male	Pre-Mesolithic		X	
8	Lower left M_3_	Lingual supragingival	18–35	Male	Neolithic	(P)	X	
9	Lower left M_3_	Lingual supragingival	35+	Female	Neolithic		X	
10–1	Lower right M_2_	Lingual supragingival	18–25	Female	Neolithic	(P)	X	X
93	Lower right M_1_ + M_3_	Lingual supragingival	26–35	Female	Neolithic	Eight type 2. Seven granules cracked (P)	X	
96	Upper left M^3^	Buccal supragingival	36–45	Female	Neolithic	17 granules. Type 2 x15 granules, type 3 x2 granules, 14 granules show diagenetic effects (P)	X	
103	Lower right M_3_	Buccal supragingival	45+	Female	Neolithic	One type 2. Undamaged (P)	X	X
104	Lower left M_3_	Lingual + Mesial supragingival	45+	Female	Neolithic	Six, type 2. One cracked (P)		
47	Upper right M^3^	Distalsupragingival	+45	Female	Meroitic		X	
74	Lower right M_2_	Lingual supragingival	18–35	Female	Meroitic	(P)	X	X
106	Lower left M_2_	Buccal supragingival	36–45	Male	Meroitic	Six damaged, one type 1. Five type 2, of which one cracked. One type 3 (P)	X	X

Preliminary analysis of excavated skeletons is conducted on site. The human remains are then exported to the UK, where detailed bioarchaeological analysis is conducted. The dentitions of the Al Khiday 2 individuals exhibit a wide range of dental pathologies such as caries, periapical lesions, ante-mortem tooth loss, enamel hypoplasia and abundant deposits of dental calculus [13]. The higher than expected rate of caries in the pre-Mesolithic populations (5%) is thought to be linked to the heavy wear that in many cases has exposed the tooth pulp and therefore damaged the structural integrity of the tooth. As expected, the cultural group that is believed to have practiced large-scale agriculture, the late Meroitic, had the highest rate of dental calculus at Al Khiday. However, one longstanding puzzle within the region has been the unexpectedly low frequency of caries among the Meroitic populations of Al Khiday (0.8%), and the site of Gabati to the north (1.3%) [14].

Samples of dental calculus were taken from 19 individuals (Table 1). Where sample size permitted, these were split into two. One half was degraded to extract microfossils, while 14 samples were analysed by sequential thermal desorption-gas chromatography-mass spectrometry (TD-GC-MS) and pyrolysis-gas chromatography-mass spectrometry (Py-GC-MS) (Information S1). This technique facilitates the identification of both free/unbound and bound/polymeric organic components [7] (Information S1. Methods and TD/Py-GC-MS detailed results). Sixteen samples were examined by light microscopy to investigate organic and inorganic micro-debris that had become entrapped in the calculus matrix. The extraction of microfossils was conducted according to a standard method [3] [7]. Samples were first rinsed in 0.6 M HCl for 5 min to remove adhered surface carbonates, dried and coarsely ground. The resulting powder was suspended in 1.5 ml of 0.6 M HCl, vortexed every 15 minutes for 1 hour, then centrifuged at room temperature at 13,000 rpm for 15 minutes. An Olympus IX 71 inverted microscope was used at between 50–200 magnifications for viewing and imaging was conducted using a Colour View camera and Cell D imaging system.

## Results

Of the 14 samples examined using TD-GC-MS and Py-GC-MS, seven produced a significant or moderate amount of organic material, the remaining samples produced very little (Information S1. Methods and TD/Py-GC-MS detailed results). Microfossils were encountered in all but two of the samples (Table 1).

### Cooking and heating

**Figure 2:**
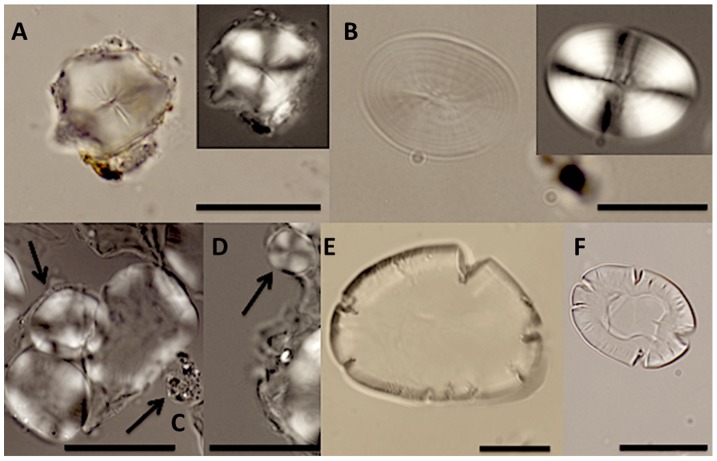
Starch granules extracted from dental calculus. (A) grave 11, polyhedral starch granule *type 1.* Superficially similar to *Cyperus rotundus* L. (note the small flecks of adhering calculus around the edge), (**b**) grave 104, oval starch granule *type 2*, (**c**) grave 96, partially degraded *type 2* starch granules, swollen and with extinction cross losing clarity. A smaller swollen granule is also lodged in the remains of the cellular wall (top left arrow), and a fleck of soil (bottom right arrow), (**d**) grave 96, small round *type 3* starch (arrow), with a part of larger *type 2* starch nearby, both starch granules are still partially embedded in dental calculus matrix, (**e** and **f**) grave 93, two starch granules in which morphology has been lost due to processing and/or cooking, note the large cracks at their margins. Scale bars are all 20 microns.

Evidence for cooking and smoke inhalation was identified in all samples through the pyrolysate total ion chromatograms (TICs) which identified components indicative of ‘black carbon’, i.e. charcoal or soot [15] [16] [17] [18], with a correlation between the amount of ‘free’ (volatile) organic material revealed by TD-GC-MS and the ‘bound’ char observed in the Py-GC-MS. Benzene was the major compound present in the pyrolysates of all but one sample (∼7–56% of total quantified pyrogram peak area), along with significant amounts of toluene (∼6–23%) and moderate quantities of ethyl benzene and o-, m- and p-xylenes (∼1–8% total). More notable were significant amounts of naphthalene, methyl naphthalene, biphenyl and methyl biphenyl, also typical of chars [15] [16] [17] [18]. The protein marker benzonitrile [19] was present in low to moderate abundance, the mean of ∼11% fairly typical of previously characterised protein-containing chars [15] [16] [17] [18]. The presence of the main combustion markers fluoranthene and pyrene, along with phenanthrene, supports the evidence for expose to fire/cooking and is consistent with the ‘char’ markers observed in the pyrogram. Variation observed in the amount of protein markers, including benzonitrile, which suggests that protein intake throughout the period of occupation of the site was highly variable between individuals, regardless of the chronological period, is notable. Although the interpretation must be somewhat tentative, it is interesting that the total hydrocarbon ‘char’ markers in the Neolithic samples is higher (mean 66%) than either the Pre-Mesolithic (mean 59%) or Meroitic (mean 41%). Light microscopy yielded a variety of organic and inorganic micro-debris, including starch granules, plant fibres and micro-charcoal in all the samples. The starch granules formed three distinct morphological groups suggestive of at least two different plant origins (Information S2. Starch granule morphology and general distribution of types). In the pre-Mesolithic samples, most granules (n = 17) are small (18–25 µm) and sub-polyhedral to polyhedral with a central hilum (*type 1*) (Figure 2). All these starch granules appear undamaged. In some cases starch granules occurred in groups of two or three, still intact and lodged within remains of the thin cellular wall (Figure 2). This suggests little or no external processing. A smaller number (n = 3) of larger oval granules with visible thin lamellae and clear extinction crosses were also present. No diagenetic effects [20] are apparent and the granules display no evidence of any form of processing or heating either in the presence of water (which leads to swelling) or roasting (which leads to drying and cracking); this suggests the plant food may have been ingested raw or after only little heating [21]. In the Neolithic samples, only the larger oval granules were present (n = 25) (*type 2*) (Figure 2). Here though, the granules show evidence of alteration indicative of heating, even though in some cases they are still contained in their cellular matrix. In one sample (burial 96) (Figure 2), all the starch granules are uncracked, slightly swollen and with a reduced birefringence, suggesting incipient gelatinization. However, they were sufficiently intact and morphologically consistent to suggest they have a different plant origin.

Granules in two of the remaining three Neolithic samples (burials 93, 104) (Figure 2) are almost all enlarged and cracked which suggests grinding and/or exposure to dry heat, such as roasting. This ties in well with the increased chemical evidence for exposure to fire in the later periods. A third morphological type consisting of smaller round starch granules (*type 3*), was also observed in low numbers in the Neolithic and Meroitic samples. In one case one *type 2* and one *type 3* lightly swollen granules were found in association embedded in the remains of the cellular wall (Figure 2). Only one Meroitic sample was examined for microfossils and here the granules were principally *type 2* granules; though a small number of *types 1* and *3* granules were also present, these granules were undamaged (Information S2. Starch granule morphology and general distribution of types).

### Cyperus rotundus

**Figure 3:**
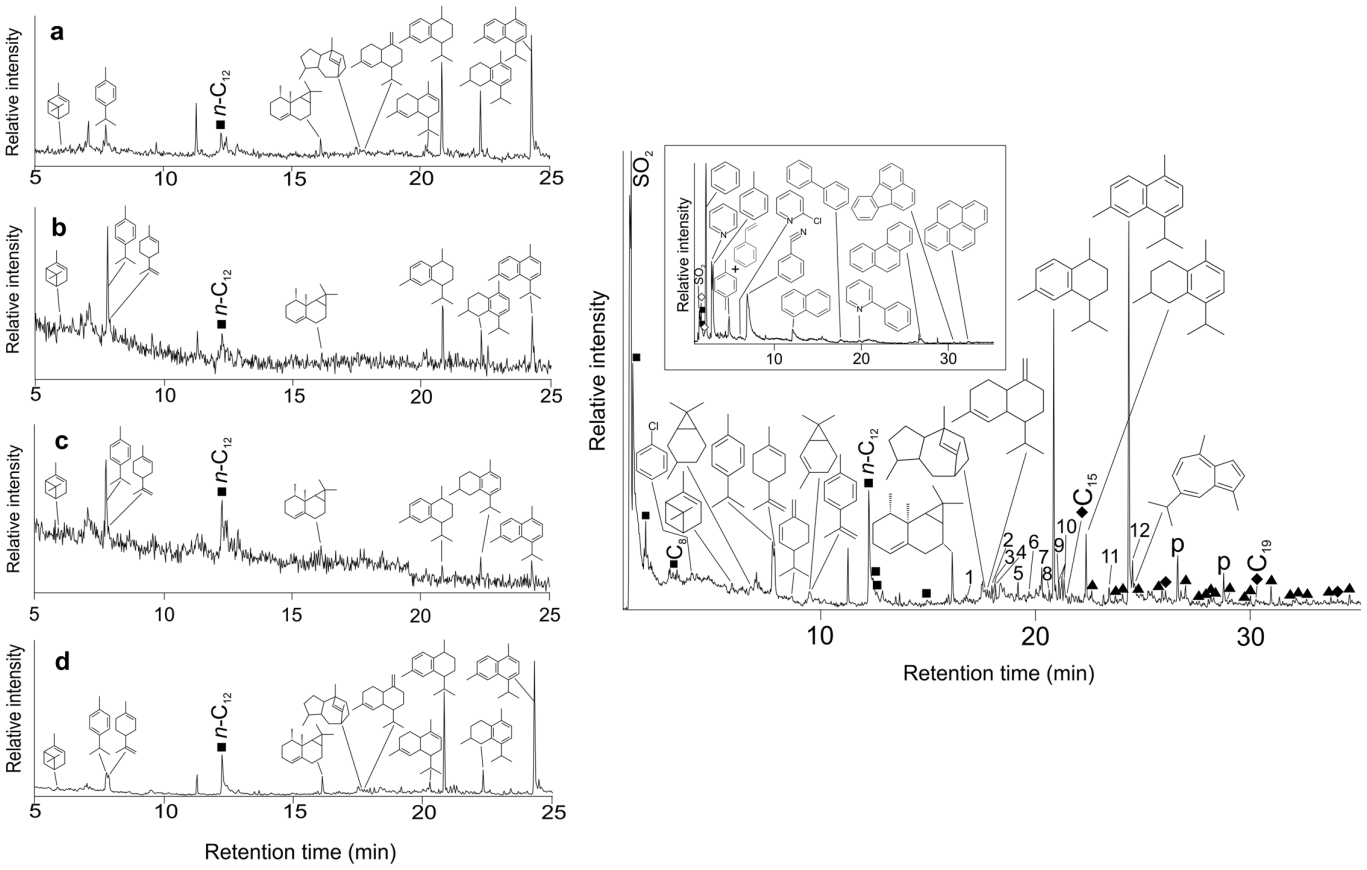
Reconstructed total ion chromatogram of the thermal desorption profiles (310°C for 10s) of human calculus samples. **Figure 3a.** (A) Pre-Mesolithic Burial 35, (b) Neolithic Burial 10I, (c) Neolithic Burial 103 and (d) Meroitic Burial 74. The structures of the terpenoid compounds characteristic of *C. rotundus* are shown, i.e. the main monoterpenoid compounds identified: α-pinene, *p*-cymene and limonene, and the main sesquiterpenoid compounds identified: calarene (β-gurjunene), rotundene, γ-muurolene, α-muurolene, calamenene, calamene and cadalene. The filled square, *n*-C_12_ indicates dodecene (see text). **Figure 3b** **.** Reconstructed total ion chromatogram of the thermal desorption profile (310°C for 10s) of human calculus, Burial 74, 5.46 mg. Peak identities (x indicates carbon chain length): filled squares, Cx indicates alkenes; filled circles; filled triangles indicates C_16_ - C_23_ methyl, ethyl- and butyl- branched alkanes; filled diamonds, Cx indicates alkylcyclohexanes. Also shown are the structures of chlorobenzene, seven monoterpenoid compounds identified: α-pinene, trans-carane, *p*-cymene, limonene, β-phellandrene, 2-carene and *p*-cymenene, and seven sesquiterpenoid compounds identified: calarene (β-gurjunene), rotundene, γ-muurolene, calamenene, calamene, cadalene and guaiazulene. In addition, sequiterpenoid compounds numbered 1 to 12 were identified as: 1 =  norrotundene, 2 = α -copaene, 3 =  cubinene (cadina-1,4-diene), 4 =  α-cedrene, 5 =  unidentified sesquiterpenoid, 6 =  γ-selinene, 7 =  α-muurolene, 8 =  γ-cadinene, 9 =  α-cadinene, 10 =  calacorenes (×3), 11 =  dehydrocadalene, 12 =  an isomer of cadalene. SO_2_ indicates sulphur dioxide. Inset displays a reconstructed total ion chromatogram of the pyrolysis profile (610°C for 10 s) of this sample, after thermal desorption (310°C for 10 s). Peak identities: filled squares, Cx indicates alkenes, open diamonds indicates propenenitrile and butenenitrile. Also shown are the structures of ten aromatic compounds identified: benzene, pyridine, toluene, styrene, *p*-xylene (coeluting with styrene), 2-chloropyridine, benzonitrile, naphthalene, biphenyl and 2-phenylpyridine, and three polynuclear aromatic hydrocarbons: phenanthrene, fluoranthene and pyrene. SO_2_ again indicates sulphur dioxide.

The chemical profiles in burials 35 (pre-Mesolithic), 10-I (Neolithic), 103 (Neolithic) and 74 (Meroitic) point predominantly and specifically to *Cyperus rotundus* [22] [23] [24] (Information S1), (Figure 3) while burials 41 and 64 (both pre-Mesolithic) also have tentative evidence for *C rotundus*. *Cyperus rotundus* is therefore represented in dental calculus samples from all periods, representing a time span of more than 7000 years.

The chemical evidence for *C. rotundus* is most clearly demonstrated in the Meroitic burial 74 (Figure 3) with the identification of a number of characteristic mono- and sesquiterpenoids, including norrotundene and rotundene (Figure 3b) (Information S1. Methods and TD/Py-GC-MS detailed results, Table 2), which were present in minor abundance, these same terpenoids having been previously identified in minor to moderate abundance in the essential oil of the *C. rotundus* rhizome/tuber [23] [24] (Information S1. Methods and TD/Py-GC-MS detailed results). These minor *C. rotundus* components were not detected in burials 10-I, 103 due to the relatively small amount of organic material present in these samples (Figure 3a). Although calamene, calamenene and cadalene, identified as significant components in these samples, are known to be constituents of the essential oil of fresh *C. rotundus* rhizome/tuber, their potential origin as diagenetic products from other more labile sequiterpenoids such as cadinenes must also be considered. However, the presence of calarene (β-gurjunene) in all four samples, which would not be a product of diagenesis and is known to be present in the essential oil component of *C. rotundus* in minor to moderate abundance [23] [24] (Information S1. Methods and TD/Py-GC-MS detailed results), together with the suite of monoterpenoid and sesquiterpenoids identified in the calculus samples and previously observed in the rhizomes/tubers of *C. rotundus* [22] [23] [24] (Information S1. Methods and TD/Py-GC-MS detailed results), is indicative of this plant species in these samples. It should also be noted that the lack of oxygenated mono- and sesquiterpenoids normally present in *C. rotundus* reflects the bio-transformations in the mouth as a result of human oral bacteria [7]. Notably, dialkyl branched alkanes were identified in samples 35, 10-I, 103 and 74, dominated by the 5,5-diethylalkanes, in addition to lesser amounts of 3,3-diethyl-, 3-ethyl-3-methyl, 5,5-dibutyl-, 5-butyl-5-ethyl- and 6,6-dibutyl-alkanes [24] (Figure 3) (Information S1. Methods and TD/Py-GC-MS detailed results). These methyl, ethyl and butyl branched alkanes of C_15_ to C_23_ are indicative of microorganisms [24] [25] (Information S1. Methods and TD/Py-GC-MS detailed results). Their association with the *C. rotundus* terpenoids, combined with the information outlined above suggests that they most likely derive from a microorganism associated with the tubers and rhizomes, or the immediate environment in which they grew.

Given the chemical evidence for ingestion of *C. rotundus*, the starch granules were compared to modern *C. rotundus* L. reference material. There is a tentative morphological correlation between the modern reference material and the starch granules in the pre-Mesolithic samples, though modern examples from the Near East appear more rounded and lack fissures emerging from the hilum. Identification of the botanical origin of starch granules is challenging, however. The general morphology of the *type 1* granules is also reminiscent of starch granules found in certain tribes of the Poaceae family, such as Paniceae and Andropogoneae [26]. For example some species of the genus *Setaria* have starch granules that are morphologically similar with a fissured hilum, often stellate, though slightly smaller. A large number of species of these tribes are gathered still today in many regions of Africa [27]. It is therefore currently not possible, to provide a secure provenance for these starch granules.

Though most of the starch granules from the Neolithic samples have been altered through heating and their original shape is very likely to have been modified, the possible association and morphology of large (*type 2*) and small (*type 3*) granules in one instance is reminiscent of the bimodal distribution that exists in the C_3_ Triticeae tribe of the Poaceae Family [28].

### Other evidence

Some of the plant fibres were similar to those found in the stems of monocotyledon plants. These remains were particularly abundant in the pre-Mesolithic and Neolithic individuals and could suggest an extra-masticatory use of teeth, as a third hand, to process plant material for use in items of material culture. Inorganic debris, including soil flecks, quartz and other mineral grit was ubiquitous. There are many ways dirt could be accidentally ingested including on dirty food items, or while using teeth to process plant material.

### Environment

With regards to environmental reconstruction, it is notable that the monoterpene, limonene, is absent in the pre-Mesolithic suite of *C.rotundus* terpenoids, yet there is an increasing relative abundance of this terpenoid in the Neolithic (1–2% of volatile thermal extract) and Meroitic (6% of volatile thermal extract) samples. This correlates with previous studies on terpenes in plants where the abundance of this monoterpene increases with a decrease in humidity and precipitation [29]. The presence of organochlorine compounds within the dental calculus, and the generation of sulphur dioxide on heating, tentatively presumed to come from lignosulphonates present [32], may suggest a saline lake/swamp containing a significant amount of sulphate (possibly in the form of gypsum); microorganisms such as bacteria, algae and fungi are known to produce naturally occurring organochlorine compounds in such environments [33] (Information S1. Methods and TD/Py-GC-MS detailed results). A group of small lakes existed in the area around Al Khiday between 7000 to 8500 years ago [34]. Buried saline lakes containing gypsum are also known to underlie this part of central Sudan [35], there is evidence of a palaeo-swamp/lake at the Al Khiday site [12] and the saline Soba area is also nearby [36]. *C. rotundus* is relatively salt tolerant [37], and would be expected to inhabit lake and swamp margins. Its rhizomes also contain a source of lignin which would allow the formation of lignosulphonates in a relatively moist, sulphate-rich environment, all of which is consistent with the findings presented here.

## Discussion

The biomolecular reflection of the environment suggests it becomes increasingly arid from pre-Mesolithic to Meroitic. This mirrors the previous stable isotope and palaeoenvironmental climate studies [12] [30] [31], with the prevailing humid/wet environment during the pre-Mesolithic. This correlates with the absence of the environmentally determined terpenoid during the pre-Mesolithic period.

The evidence extracted from the dental calculus has shown the use of fire, and possibly smoke, in all periods. Cooking on an open fire does not always fully gelatinize starch granules (Information S3. Variable gelatinization of starch granules following open fire cooking). The Hadza, for example, are known to cook their tubers for a very short time, possibly to facilitate peeling and chewing, while leaving the interior of their food raw [38]. Therefore, despite the raw appearance of the starch granules in the pre-Mesolithic samples, they could have come from food items that had been lightly heated. Cooking is further supported by the higher amount of total ‘char’ markers observed in the Neolithic samples compared with the Pre-Mesolithic and Meroitic samples. Some of the ‘char’ observed in the calculus samples may also derive from exposure to fires for non-culinary purposes. However, the higher total ‘char’ for the Neolithic period is consistent with the evidence from the microscopy for notable use of cooking at this time. The chemical data may also suggest that there was less use of fires during the Meroitic than either the Pre-Mesolithic or the Neolithic, perhaps as a result of a warmer climate at Al Khiday. The chemical data also correlates with less evidence for cooking following the Neolithic, and it is perhaps noteworthy that the calculus samples containing the most organic material were Pre-Mesolithic and Meroitic. The greater evidence for cooking in the Neolithic may have resulted in poorer survival of the organic material consumed.

*Cyperus rotundus* is particularly interesting as it is present in all periods. *C.rotundus* or ‘purple nut sedge’ is a C_4_ plant that is profligate in moist tropical environments. It has been called the ‘world’s most expensive weed’ [39] due to its ability to spread rapidly through its underground storage system of bulbs, rhizomes and tubers, whose proliferation may be caused by an excess of carbohydrates [40]. *C. rotundus* was highlighted as a potentially key component of the diet of the Late Palaeolithic population of Wadi Kubbaniya in southern Egypt (17,000–15,000 BC) 1000 km north of Al Khiday, where it predominated in the abundant assemblages of charred plant remains [41]. However, despite identification of several plant species in charred human coprolites, *C. rotundus* was not detected [41].

Chewing, followed by expulsion of pithy quid, is common among traditional tuber-eaters such as the Hadza [42] even after these have been cooked [43]. *C. rotundus* tubers can be pithy and expelling the quid after chewing may explain why no physical evidence for *C. rotundus* was found in the coprolites at Wadi Kubbaniya despite the abundant carbonised remains.

The use of *C. rotundus* as a carbohydrate staple is documented across tropical regions among recent hunter-gatherers and as a famine food in some agrarian societies; its nutritional value is enhanced by the presence of lysine, an essential amino acid [41]. *C. rotundus* has also been considered as part of a package of high starch, tuber-rich sedges that may have been exploited by Pliocene hominins [44] [45]. Though today it is considered to have a bitter taste [41], *C. rotundus* was one of three tuber staples among Aboriginal populations in Central Australia [46]. While the tubers can be small and time-consuming to harvest, experimental harvesting recovered over 21,000 tubers per m^2^ in permanently wet environments; in drier areas, although the quantity decreased, tuber size increased and bitterness was diminished [41]. The availability of other, possibly better tasting C_3_ plants, most likely cultivated crops, in the Neolithic and Meroitic periods begs the question of why *C. rotundus* continued to be ingested.

In addition to its value as a source of carbohydrates, *C. rotundus*, has many other qualities that have been widely recognised. Numerous accounts of the non-nutritional use of *C. rotundus* from ancient Egypt [47], Mycenean Greece [48] and elsewhere exist, including its use for aromatic purposes and in water purification [41]. *C. rotundus* is mentioned by the Hippocratic doctors (5th century BC), Theophrastus, Pliny and Dioscorides (1st century AD), as a source of perfume and medicine [49]. Dioscorides also highlights the use of *C. rotundus* tubers as an ingredient of ancient Egypt's best known perfume, kuphi or kyphi, an incense that also had medicinal properties and provides a preparation to perfume goose or pork fat made by mixing *C. rotundus* with other vegetable agents [49]. A wide range of medicinal uses have been recorded [22] [50] [51] [52] [53] [54] [55] and anti-microbial [56] [57], anti-malarial [58], anti-oxidant [59] [60] [61] [62] and anti-diabetic [63] compounds have been isolated and identified. Tubers are still used today in herbal medicine in the Middle East, Far East and India [48], for perfume and animal fodder [51] [64] and as incense in Burkina Faso [65].

*C. rotundus* tubers are very likely to have been eaten principally for their nutritious qualities during pre-Mesolithic periods; however, their continued use in agricultural periods suggests they may also have been used for other purposes, instead of, or in addition to their value as a nutritional resource. Though the ingestion of plants specifically for medicinal purposes is now accepted among higher primates [66], demonstrating similar behaviour among early human populations is challenging [7] [67]. However, the non-nutritional qualities of *C. rotundus* suggest that it could have been appreciated for its aromatic or medicinal qualities in addition to its potential value as a lean period or fall-back food.

The development of dental caries is strongly associated with diet, most notably the presence of sugars including fermentable carbohydrates which interact with plaque bacteria to cause demineralisation; the presence of caries also increases with age (68). At the late Palaeolithic site in Taforalt, Morocco [69] a link has been made between specific highly starchy cariogenic foodstuffs found at this site, the time period of expansion of *Streptococcus mutans* which is a leading contributor of tooth decay today, and the unexpectedly high prevalence of caries in teeth, to suggest that the food items ingested caused the high caries rate found in the population here. Laboratory testing of *C. rotundus* extract has demonstrated that this inhibits *S. mutans* [70] [71]. As the type of food ingested can have a direct effect on the health of teeth (68), we suggest that chewing *C. rotundus* tubers may have contributed to the unexpectedly low prevalence of dental caries in the Meroitic samples at Al Khiday and possibly also Gabati.

The chemical evidence from the ‘char’ compounds and combustion markers (polynuclear aromatic hydrocarbons; PAHs) confirm that cooking and exposure to smoke is present in all the samples for which there were results. Fire is likely to have been used in a wide range of circumstances, including for cooking, protection and warmth, and smoke has many uses, for example as an insect repellent, in food preservation and for communication as well as some raw material preparation. The diet is likely to have included a wider range of foods than that identified in this study due to diagenetic processes preferentially preserving or degrading biomolecules in the food originally trapped in the calculus.

Biomolecular studies of dental calculus are highly challenging, as the organic material entrapped is variable and the quantities are often small, yet despite this, the study presented here has not only detected and identified a wide range of organic compounds in the samples analysed, but has also permitted the identification of *C. rotundus*. These results highlight the potential for future biomolecular studies which complement ongoing research focussed on the more labile biomolecule of DNA.

The evidence from a growing number of pre-agrarian sites [41] [68] [72] [73] for use of locally available wild plants, suggests broad ecological knowledge and extensive use of plants, while there is widespread ethnographic evidence for use of teeth in non-masticatory activities including holding, softening or shredding material. In archaeological contexts, physical evidence for non-masticatory use of teeth, based on tooth wear and attrition, occurs across the geographical and temporal spectra [74] [75] [76] [77] [78] [79] [80] [81]. At Al Khiday, the plant fibres found embedded in the samples of dental calculus, offer an empirical reminder of this process. There is a noticeable difference in the tooth wear between the pre-Mesolithic and Neolithic individuals who have irregular, often cupped, molar wear and the Meroitic population whose teeth have flat wear [13]. However, more numerous samples would be required to determine whether any link can be made between the nature of the plant fibre evidence and the varying tooth wear forms. Though the plant fibres cannot be identified to their source and may come from different plants, *C. rotundus* stems are known to have been used in basketry and matting.

The extensive evidence extracted from the dental calculus for the ingestion and working of plants, as well as the use of *C. rotundus* tubers as a source of carbohydrates and possibly as medicine or as flavouring, fits well within the perspective of broad environmental and ecological knowledge in prehistoric periods. Today, *Cyperus rotundus* is used as animal fodder and is considered the world's most costly weed as its prolific tubers spread underground, but while its tenacity and prolificity is problematic for farmers now[39] [40], these qualities made it an abundant and accessible resource in the past.

The development of studies on chemical compounds and microfossils extracted from dental calculus will help to counterbalance the dominant focus on meat and protein that has been a feature of pre-agricultural dietary interpretation, up until now The new access to plants ingested, which is provided by dental calculus analysis, will increase, if not revolutionise, the perception of ecological knowledge and use of plants among earlier prehistoric and pre-agrarian populations.

## Supporting Information

Information S1 **Methods and TD and Py-GC-MS detailed results.**
(PDF)

Information S2 **Starch granule morphology and general distribution of types.**
(PDF)

Information S3 **Variable gelatinization of starch granules following open fire cooking.** (a) raw seed (163 magnifications); (b) Internal structure of raw seed, (5317 magnifications); (c) partially gelatinized starch granules (5348 magnifications); (d) raw to fully gelatinized starch granules (5348 magnifications).
(PPTX)
