# Supplementary material for: Dental Calculus Reveals Unique Insights into Food Items, Cooking and Plant Processing in Prehistoric Central Sudan
Source: PLoS One. 2014 Jul 16;9(7):e100808. doi: 10.1371/journal.pone.0100808 (PMC4100759; doi:10.1371/journal.pone.0100808)
Supplement: Information S1 — Methods and TD and Py-GC-MS detailed results. (PDF) [file pone.0100808.s001.pdf]

Supplementary Information 1. Methods and TD/Py-GC-MS detailed results

TD/Py-GC-MS was performed on a CDS Pyroprobe 1000 via a CDS1500 valved interface (320°C), to a Hewlett-Packard 5890GC split injector (280°C) linked to a Hewlett-Packard 5973MSD (electron voltage 70eV, filament current 220uA, source temperature 230°C, quadrupole temperature 150°C, multiplier voltage 2200V, interface temperature 340°C). The acquisition was controlled by a HP kayak xa chemstation computer, in full scan mode (50-700amu). Approximately 2-3mg of ground sample was weighed into a quartz tube with glass wool end plugs. The tube was placed into a pyroprobe platinum heating coil and sealed into the valved interface. The sample was thermally desorbed at 310°C for 10secs in open split mode at 30mls/minute. At the same time the GC temperature programme and data acquisition commenced. Separation was performed on a fused silica capillary column (30m x 0.25mm i.d) coated with 0.25um 5% phenyl methyl silicone (DB-5). Initially the GC was held at 35°c for 5 minutes and then temperature programmed from 40°C-340°C at 6°C min and held at final temperature for 15 minutes, total 65 minutes, with Helium as the carrier gas (constant flow 1ml/min, initial pressure of 45kPa, split at 30 mls/min). The run was repeated with the same sample being pyrolysed at 610°C for 10 seconds with the split open. Peaks were identified on the basis of both their mass spectra (NIST Mass Spectral Database and additional data referenced below, and relative retention times (relative retention indices (RRIs)).

Fourteen samples of dental calculus taken from 14 individuals from Al Khiday in Central Sudan were analysed by sequential thermal desorption-gas chromatography-mass spectrometry (TD-GC-MS) and pyrolysis-gas chromatography-mass spectrometry (Py-GC-MS). This technique facilitates the identification of both free/unbound and bound/polymeric organic components [1]. Seven of the 14 calculus samples produced a significant or moderate amount of organic material, the other seven samples producing very little.

Table 1. Full results

Samples	Period	Starch granules	TD/Py-GCMS	*Cyperus rotundus*	Char/ carbonised	Dialkyl branched alkanes
11	Pre-Mesolithic	X				
12	Pre-Mesolithic	X				
35	Pre-Mesolithic	X	X	X	X	X
41	Pre-Mesolithic	X	X		X	X
55	Pre-	X				

	Mesolithic					
64	Pre-Mesolithic	X	X		X	X
77	Pre-Mesolithic	X	X		X	
88	Pre-Mesolithic	X				
111	Pre-Mesolithic		X		X	
8	Neolithic		X		X	
9	Neolithic		X		X	
10-I	Neolithic		X	X	X	X
93	Neolithic	X	X		X	
96	Neolithic	X	X		X	
103	Neolithic	X	X	X	X	X
104	Neolithic					
47	Meroitic	X	X		X	
74	Meroitic	X	X	X	X	X
106	Meroitic	X	X		X	

Burial 35 pre-Mesolithic (calculus: 0.97mg)

The thermal desorption total ion chromatogram (TIC) (Fig. 1) is dominated by a significant number of monoterpenoids (C_{10}) and sesquiterpenoids (C_{15}). Two monoterpenoids were identified, i.e. α-pinene and *p*-cymene. In contrast, the TD chromatogram revealed a complex suite of sesquiterpenoids which included, in order of retention time (volatility), calarene (β-gurjunene), rotundene, γ-muurolene, cubinene, α-cedrene, an unidentified sesquiterpenoid, cypera-2,10-diene (tentatively identified; trace), an unidentified sesquiterpenoid, γ-selinene (trace), an isomer of calamenene, α-muurolene (trace), calamenene (major component), calacorenes (x3), calamene (major component), a dehydrocadalene, cadalene (major component), an isomer of cadalene and guaiazulene. These terpenoids have previously all been found in the tubers and rhizomes of purple nut sedge, *Cyperus rotundus* and their unusual natural occurrence and their collective presence in the dental calculus strongly suggests this specific plant source (see Table 2 and main text).

Present in moderate abundance are a series of *n*-1-alkenes and *n*-alkanes, carbon numbers C_8 to C_{12}. In addition, 1-butene was the most abundant component in the TD. The 1-butene could derive from proteinaceous material in the sample [2], perhaps produced at this relatively low temperature due to matrix effects, but the presence in the TD chromatogram of the series of alkenes and alkanes is not consistent with either protein or fat/oil derived acyl lipids, and rather suggests these or their close molecular analogues in the original food consumed. Notably, the *n*-1-dodecene is appreciably more abundant than the *n*-dodecane analogue and in its hydrated form, i.e. *n*-dodecanol, is a significant constituent of *C. rotundus* essential oil component of the

tuber/rhizome [3], and so its presence is consistent with the observed terpenoids characteristic of *C. rotundus*.

A suite of branched alkanes constituted a moderate component of the free compounds identified in the TD-GC-MS. These include 5,5-diethyltridecane, 5,5-diethylpentadecane, 5,5-diethylheptadecane, 3-ethyl-3-methylpentadecane and 3-ethyl-3-methylheptadecane, 3,3-diethylpentadecane and 5,5-dibutyldodecane. These methyl, ethyl and butyl- branched alkanes of C_{17} to C_{21} are typical of microorganisms, i.e. bacteria, algae and fungi [4], [5], [6], [7], [8]. Given their association with the terpenoids it suggests that they derive from a microorganism associated with *C. rotundus* tubers and rhizomes (see main text). Sulphur dioxide was also detected, the possible significance of which is discussed in the main text. A series of alkyl cyclohexanes with C_9, C_{11}, C_{13}, C_{15} and C_{17} saturated side chains were also detected; odd carbon numbered alkyl cyclohexanes can be indicative of an algal source (their even numbered analogues are indicative of bacteria [9], which would be consistent with the lakes and swamp margins environment of *C. rotundus*, though the precise origin here is uncertain. The aromatic compounds benzene, ethyl benzene, and o-, m- and p-xylenes, were identified, but their wide natural distribution and minor abundance does not allow a meaningful assessment of their likely origin. An alkyl dihydropyran and 4,5-dihydro-2,4-dimethyl-1H-imidazole (2,4-dimethyl-2-imidazoline) were also identified, the latter previously observed in fish tissue [10] and likely to be protein derived. Sulphur dioxide was also detected and its generation at ~300°C suggests an organo-sulphate origin, possibly lignosulphonates (see main text). Sulphur dioxide is known to be a major pyrolysis product of lignosulphonates (see main text) [14] [15], and its organic nature would facilitate sulphur dioxide generation at this relatively low temperature, although this inference can be no more than speculative and further research is necessary.

The pyrolysate TIC identified components indicative of ‘black carbon’, i.e. charcoal or soot [11], [12], [13], [14]. Benzene was the major compound present in the pyrolysate (~40% of total quantified pyrogram peak area), along with significant amounts of toluene (~15%) and moderate quantities of ethyl benzene and o-, m- and p-xylenes (~5% total). More notable were significant amounts of naphthalene, methyl naphthalene, biphenyl and methyl biphenyl, also typical of chars [11], [12], [13], [14]. The protein marker benzonitrile [2] was present in moderate abundance (~8%) typical of previously characterised protein-containing chars [11], [12], [13], [14]. The unsaturated compounds 1-butene, 1,3-butadiene, 1,3-pentadiene and cyclopentene were present in significant abundance in the char, although their origin in this context is uncertain. No

carbohydrate markers indicative of starch [15] could be detected, which may suggest diagenetic changes given the resilient biomarkers characteristic of *C. rotundus* present in the TD-GC-MS and the known susceptibility of carbohydrates to degradation (carbohydrates>DNA >proteins>lipids). The presence of the main combustion markers fluoranthene and pyrene, along with phenanthrene, supports the evidence for cooking/expose to fire in this sample and is consistent with the 'char' markers observed in the pyrogram. Sulphur dioxide observed in the TD was also present in the pyrolysate, which may be due to matrix effects in the dental calculus samples. Its likely origin is the same as that for the gas generated in the TD.

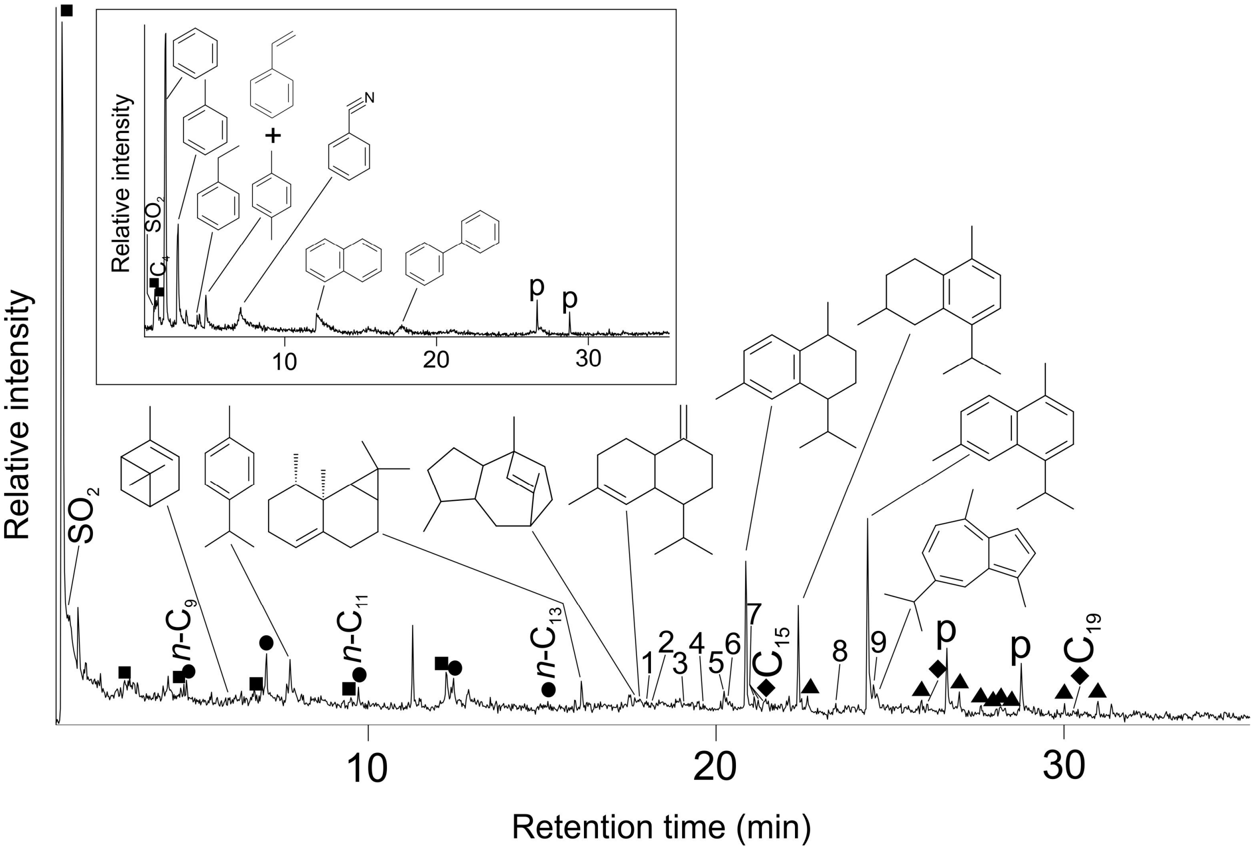

Figure S1 Reconstructed total ion chromatogram of the thermal desorption profile (310ºC for 10s) of human calculus, Burial 35, 0.97mg. Peak identities (x indicates carbon chain length): filled squares, Cx indicates alkenes; filled circles, Cx indicates alkanes; filled triangles indicates C_{17} - C_{21} methyl, ethyl- and butyl- branched alkanes; filled diamonds, Cx indicates alkylcyclohexanes. Also shown are the structures of two monoterpenoid compounds identified: α-pinene and *p*-cymene, and seven sesquiterpenoid compounds identified: calarene (β-gurjunene), rotundene, γ-muurolene, calamenene, calamene, cadalene and guaiazulene. In addition, sequiterpenoid compounds numbered 1 to 9 were identified as: 1 = cubinene (cadina-1,4-diene), 2 = α-cedrene, 3 = cypera-2,10(?)-diene (tentatively identified), 4 = γ-selinene (trace),

5= an isomer of calamenene, 6 = α-muurolene (trace), 7 = calacorenes (×3), 8 = dehydrocadalene, 9 = an isomer of cadalene. SO_2 indicates sulphur dioxide. Inset displays a reconstructed total ion chromatogram of the pyrolysis profile (610ºC for 10s) of this sample, after thermal desorption (310ºC for 10s). Peak identities: filled squares, Cx indicates alkenes. Also shown are the structures of eight aromatic compounds identified: benzene, toluene, ethyl benzene, styrene, *p*-xylene (coeluting with styrene), benzonitrile, naphthalene and biphenyl. SO_2 again indicates sulphur dioxide. (p = pyroprobe contaminants)

Table 2. *Cyperus rotundus* tubers /rhizomes compounds present in the samples

**Compound**	***Cyperus rotundus*†**	**Burial 35 Pre-Mesolithic**	**Burial 10-I Neolithic****	**Burial 103 Neolithic****	**Burial 74 Meroitic**
α-pinene	√	√	√	√	√
p-cymene	√	√	√	√	√
limonene	√	- ‡	√	√	√
α-phellandrene	√	-	-	-	-
β-phellandrene	-	-	-	-	√
p-cymenene	√*	-	-	-	√
n-dodecanol/*n*-dodecene	√	√	√	√	√
calarene (β-gurjunene)	√	√	√	√	√
norrotundene	√	-	-	-	√
rotundene	√	√	-	-	√
γ-muurolene	√	√	-	-	√
cubinene	√	√	-	-	√
α-copaene	√	-	-	-	√
α-cedrene/cedrol*	√*	√	-	-	√
selinene (β- or γ)	√	√	-	-	√
α-muurolene	√	√	-	-	√
γ-cadinene	√	-	-	-	√
δ-cadinene	√	-	-	-	-
calamenene	√	√	√	√	√

α-cadinene	-	√	-	-	√
α-calacorene	√	-	-	-	√
β-calacorene	√	√	-	-	√
γ-calacorene	√	-	-	-	√
calamene	√	√	√	√	√
a dehydrocadalene	?	√	-	-	√
cadalene	√	√	√	√	√
an isomer of cadalene	?	√	-	-	√
guaiazulene	?	√	√	-	√

†present in tuber and rhizome

‡absent due to prevailing humid/wet environment

*previously identified in *Cyperus* genus, i.e. *C. articulates*

**relatively little organic material

Burial 41 pre-Mesolithic (calculus: 0.86 and 4.55mg)

The thermal desorption total ion chromatogram (TIC) revealed relatively few detectable components, with the exception of 1-butene which dominated the chromatogram, possibly deriving from lipid or proteinaceous material in the sample [2], and perhaps produced at this relatively low temperature due to matrix effects. Present in minor abundance are a series of *n*-alkanes, carbon numbers C_9 to C_{12} and C_{14}. Their presence in the TD chromatogram, without their *n*-1-alkene counterparts observed in sample 35, is not consistent with either protein or fat/oil derived acyl lipids, and suggests these or their close molecular analogues derive from the original food consumed, (e.g. fruit or plant waxes). The unsaturated cyclic compounds cyclohexene and methylcyclohexene were also observed, possibly suggesting a polyunsaturated lipid component, generated at this relatively low temperature due to matrix effects caused by the mineral component in the dental calculus. Minor amounts of benzene and toluene were also detected, as was the monoterpenoid *p*-cymene (see discussion).

The pyrolysate TIC identified components indicative of 'black carbon', i.e. charcoal or soot [11], [12], [13], [14]. Benzene was the major compound present in the pyrolysate (~37% of total quantified pyrogram peak area), along with significant amounts of toluene (~17%) and moderate

quantities of ethyl benzene and o-, m- and p-xylenes (~8% total). More notable were significant amounts of naphthalene, methyl naphthalene, biphenyl and methyl biphenyl, also typical of chars [11], [12], [13], [14]. In marked contrast to the previous sample (35), the protein marker benzonitrile [1] was present only in very minor abundance (~0.3%), possibly indicative of a low protein-containing char [11], [12], [13], [14] and diet for this individual. The unsaturated compounds 1-butene, 1,3-butadiene, penta-1,3-diene and cyclopentene were present in moderate abundance, although their origin in this context is uncertain. No carbohydrate markers indicative of starch [15] could be detected. The presence of the main combustion markers fluoranthene and pyrene, along with phenanthrene, supports the evidence for cooking/expose to fire in this sample and is consistent with the 'char' markers observed in the pyrogram. Sulphur dioxide was identified in the pyrolysate, which suggests a lignosulphonate origin (see main text), although this inference can be no more than speculative and further research is necessary.

Burial 64 pre-Mesolithic (calculus: 5.32mg)

The thermal desorption total ion chromatogram (TIC) revealed relatively few detectable components, with the exception of 1-butene which dominated the chromatogram, possibly deriving from lipid or proteinaceous material in the sample [2], and perhaps produced at this relatively low temperature due to matrix effects. Present in minor abundance are a series of *n*-1-alkenes, carbon numbers C_5 to C_8, their presence in the TD chromatogram suggests acyl lipids from an oxidised and/or degraded fat/oil. The C_{12} alkene is also observed, although its presence here, with the monoterpenoid *p*-cymene in similar abundance, suggests a non-lipid source (see discussion). Minor amounts of benzene, toluene and ethylbenzene were also detected, as was the halogenated compound, chlorobenzene. This last compound is known to occur in microorganisms exposed to a saline environment (see main text). Also tentatively identified as a minor constituent, was tetrahydro-3,6-dimethyl-2H-pyran-2-one; this can be a carbohydrate pyrolysis product [16], although in the absence of any other carbohydrate markers its significance is difficult to determine.

The pyrolysate TIC identified components indicative of 'black carbon', i.e. charcoal or soot [11], [12], [13], [14]. Benzene was the major compound present in the pyrolysate (~45% of total quantified pyrogram peak area), along with moderate amounts of toluene (~6%) and minor quantities of ethyl benzene and o-, m- and p-xylenes (~1% total). Notable were moderate amounts of naphthalene, biphenyl and methyl biphenyl, also typical of chars [11], [12], [13],

[14]. The protein marker benzonitrile [2] was present in significant abundance (~16%) fairly typical of previously characterised protein-containing chars [11], [12], [13], [14], although this and the presence of pyridine (1% of total quantified pyrogram peak area), another protein marker [2], it could be indicative of a relatively higher protein diet compared to other individuals at Al Khiday. The unsaturated compounds 1-butene and penta-1,3-diene were present in minor abundance, although their origin in this context is uncertain. No carbohydrate markers indicative of starch [15], and no polynuclear aromatic hydrocarbons (PAHs) indicative of combustion could be detected. Sulphur dioxide was identified in the pyrolysate, which suggests a lignosulphonate origin (see main text), although this inference can be no more than speculative and further research is necessary.

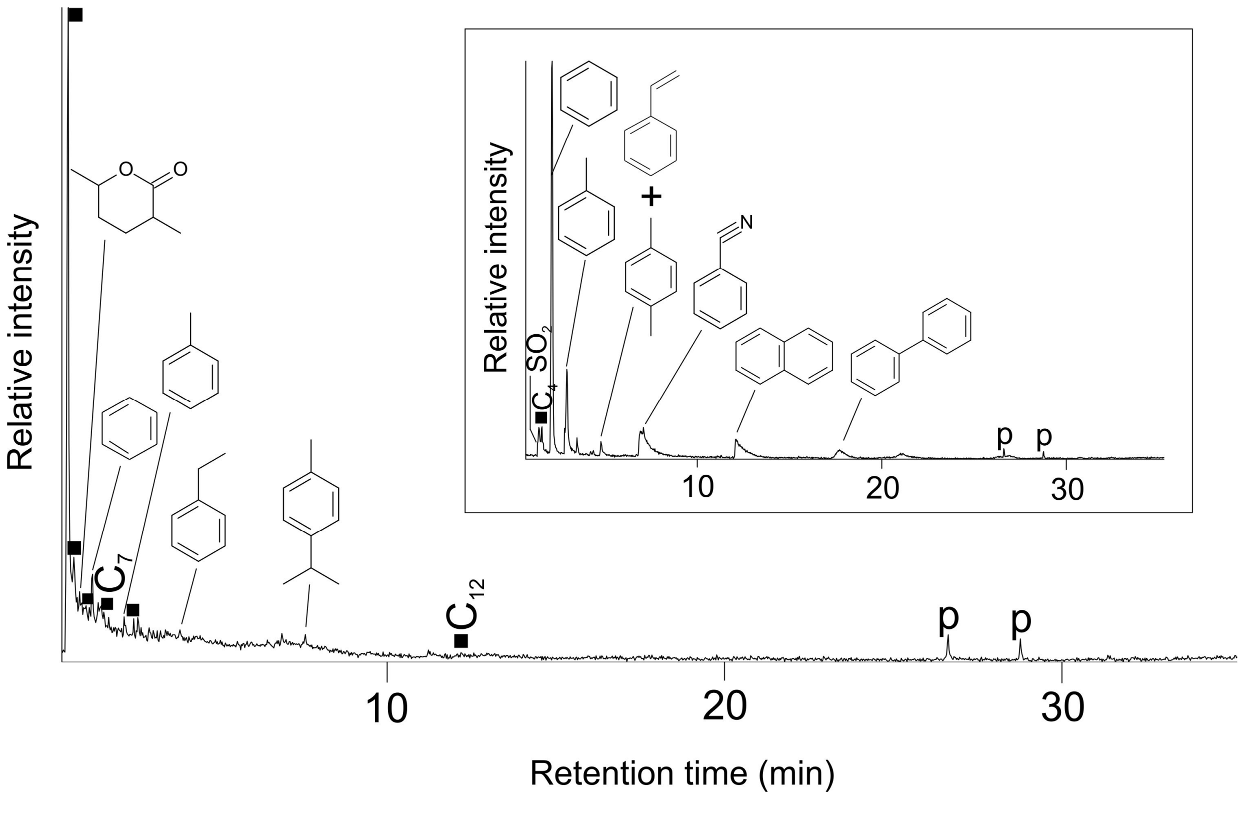

Figure S2 Reconstructed total ion chromatogram of the thermal desorption profile (310ºC for 10s) of human calculus, Burial 64, 5.32mg. Peak identities (x indicates carbon chain length): filled squares, Cx indicate alkenes. Also shown are the structures of a pyran and four aromatic compounds identified: tetrahydro-3,6-dimethyl-2H-pyran-2-one (tentatively identified), benzene, toluene, ethyl benzene and the monoterpenoid, *p*-cymene. Inset displays a reconstructed total ion chromatogram of the pyrolysis profile (610ºC for 10s) of this sample, after thermal desorption (310ºC for 10s). Peak identities: filled squares, Cx indicates alkene. Also shown are the

structures of seven aromatic compounds identified: benzene, toluene, styrene, *p*-xylene (coeluting with styrene), benzonitrile, naphthalene and biphenyl. SO_2 indicates sulphur dioxide. (p = pyroprobe contaminants)

Burial 77 pre-Mesolithic (calculus: 6.95mg)

The thermal desorption total ion chromatogram (TIC) revealed very few detectable components, indicating the virtual absence of any free, thermally extractable organic components in this sample. It should be noted, however, that highly polar material could have been present, which would not have successfully eluted from the column, or indeed volatilised sufficiently in the probe. Of the trace components which were present chloromethane was the major constituent, with chlorobenzene also detected. These chlorinated compounds are known to occur in microorganisms exposed to a saline environment (see main text). The only other components identified were sulphur dioxide, 1,3-pentadiene and benzene. Due to the trace quantities, no meaningful interpretation can be assigned to these constituents.

The pyrolysate TIC also contained relatively little detectable organic material, and when taken together with the TD indicates a low abundance of organic material in this calculus sample, although it should be said that this may be due to diagenetic changes. However, the components which were identified in the pyrolysate TIC were indicative of 'black carbon', i.e. charcoal or soot [11], [12], [13], [14]. Benzene was the major compound present in the pyrolysate (~48% of total quantified pyrogram peak area), along with significant amounts of toluene (~11%) and minor quantities of ethyl benzene and xylenes (~1% total). A moderate amount of naphthalene and minor amount of biphenyl were also detected, which are also typical of chars [11], [12], [13], [14]. The protein marker benzonitrile [2] was present in significant abundance (~23%) fairly typical of previously characterised protein-containing chars [11], [12], [13], [14], although this and the presence of propanenitrile, another protein marker [2], the abundance could be indicative of a relatively higher protein diet compared to other individuals at Al Khiday. The unsaturated compounds 1-butene, 1-pentene and penta-1,3-diene were present in minor abundance, although their origin in this context is uncertain. Chloromethane was also detected, albeit as a minor constituent. It is known to occur in microorganisms exposed to a saline environment (see main text).

Burial 111 pre-Mesolithic (calculus: 1.59mg)

The separate step of thermal desorption was not carried out on this sample, so it underwent pyrolysis only at 610°C. The TIC identified few organic components. Of those identified, benzene (~42% of total quantified pyrogram peak area), toluene (~14%) and xylenes (3%) are indicative of 'black carbon', i.e. charcoal or soot [11], [12], [13], [14]. Also notable are furan (~24% of total quantified pyrogram peak area) and acetone (~9%), which are pyrolysis carbohydrate markers [2] and possibly indicative of a significant carbohydrate component. The aliphatic hydrocarbons 1-butene, 1-pentene, 1-hexene and n-hexane (total ~8% of total quantified pyrogram peak area), possibly suggest a polyunsaturated lipid component in this sample.

Burial 8 Neolithic (calculus: 1.26mg)

The thermal desorption total ion chromatogram (TIC) revealed very few detectable components, indicating the virtual absence of any free, thermally extractable organic components in this sample. It should be noted, however, that highly polar material could have been present, which would not have successfully eluted from the column, or indeed volatilised sufficiently in the probe. Of the trace components which were present 1-butene and chloromethane were the major constituents, with chlorobenzene also detected. These chlorinated compounds are known to occur in microorganisms exposed to a saline environment (see main text). The dominance of 1-butene in the TD is similar to previous samples and possibly derives from lipid or proteinaceous material in the sample [2], and perhaps produced at this relatively low temperature due to matrix effects. The monoterpenoid *p*-cymene was detected as a trace component, and although present in such low abundance, in context with the other samples analysed a tentative origin can be cautiously ascribed (see discussion). The other components identified included the minor constituent benzene, trace quantities of xylene and sulphur dioxide. Due to the minor quantities of these compounds, no meaningful interpretation can be assigned to these constituents.

The pyrolysate TIC also contained relatively little detectable organic material, and when taken together with the TD indicates a low abundance of organic material in this calculus sample, although it should be said that this may be due to diagenetic changes. However, the components which were identified in the pyrolysate TIC were indicative of 'black carbon', i.e. charcoal or soot [11], [12], [13], [14]. Benzene was the major compound present in the pyrolysate (~50% of total quantified pyrogram peak area), along with significant amounts of toluene (~10%) and minor quantities of ethyl benzene and xylenes (~2% total). A moderate amount of naphthalene and minor amount of biphenyl were also detected, which are also typical of chars [11], [12], [13], [14]. The protein marker benzonitrile [2] was present in significant abundance (~17%) fairly

typical of previously characterised protein-containing chars [11], [12], [13], [14], although its abundance here could be indicative of a higher protein diet compared to other individuals at Al Khiday. The unsaturated compound 1-pentene was present in minor abundance, though in isolation its significance is difficult to determine. Chloromethane was also detected, albeit as a minor constituent; it is known to occur in microorganisms exposed to a saline environment (see main text).

Burial 9 Neolithic (calculus: 1.92mg)

The thermal desorption total ion chromatogram (TIC) revealed few detectable components, indicating little free, thermally extractable organic components in this sample. It should be noted, however, that highly polar material could have been present, which would not have successfully eluted from the column, or indeed volatilised sufficiently in the probe. Of the components which were present, a series of n-alkanes, C_{11} to C_{16}, and methyl branched alkanes, C_{13} to C_{17}, along with a C_{18} alkene, were detected as notable, if albeit minor, constituents of the TD chromatogram. Although the branched alkanes would suggest a bacterial origin, beyond this further work would be needed in order to determine a possible origin. The unsaturated compounds 1-butene and 1,3-pentadiene were present in moderate abundance, the former possibly deriving from lipid or proteinaceous material in the sample [2], and perhaps produced at this relatively low temperature due to matrix effects. A dimethyl-2-pentene was a significant constituent of the chromatogram (10%), although its origin here is unclear. Chloromethane was also present in moderate abundance; these chlorinated compounds are known to occur in microorganisms exposed to a saline environment (see main text). Also notable is acetone (~14% of total quantified TD chromatogram peak area), which is a pyrolysis carbohydrate markers [2] and possibly indicative of a carbohydrate component and produced at this relative low temperature due to matrix effects.

The pyrolysate TIC also contained relatively little detectable organic material, and when taken together with the TD indicates a fairly low abundance of organic material in this calculus sample, although this may be due to diagenetic changes. However, the components which were identified in the pyrolysate TIC were indicative of 'black carbon', i.e. charcoal or soot [11], [12], [13], [14]. Benzene was the major compound present in the pyrolysate (~38% of total quantified pyrogram peak area), along with significant amounts of toluene (~19%) and minor quantities of xylenes (~3% total). A moderate amount of naphthalene was also detected, which is also typical of chars [11], [12], [13], [14]. The protein marker benzonitrile [2] was present in minor

abundance (~2%) typical of previously characterised protein-containing chars [11], [12], [13], [14], although its low abundance here could be indicative of a lower protein diet compared to other individuals at Al Khiday. The unsaturated compounds 1-butene and 1-pentene were present in minor abundance, although their origin in this context is uncertain. Chloromethane was detected in moderate abundance (8%); it is known to occur in microorganisms exposed to a saline environment (see main text).

Burial 10-I Neolithic (calculus: 0.92mg)

The thermal desorption total ion chromatogram (TIC) (Fig. 1) revealed a significant number of monoterpenoids (C_{10}) and sesquiterpenoids (C_{15}), albeit in minor to moderate abundance. Three monoterpenoids were identified, i.e. α-pinene, *p*-cymene and limonene (for the significance of this latter monoterpenoid, see Meroitic sample 74 and discussion). The sesquiterpenoids identified were, in order of retention time (volatility), calarene (β-gurjunene), calamenene, calamene, cadalene and guaiazulene. Again, these terpenoids have previously all been found in the tubers and rhizomes of purple nut sedge, *C. rotundus* and their unusual natural occurrence combined with their relative abundance to each other point to the same plant source identified in pre-Mesolithic sample 35 (see Table 2 and main text).

The unsaturated compounds 1-butene and 1,3-pentadiene were present in moderate and significant abundance respectively, the former possibly deriving from lipid or proteinaceous material in the sample [2], and perhaps produced at this relatively low temperature due to matrix effects. A dimethyl-2-pentene and a dimethyl-2-hexene were significant constituents of those observed, although their origin here is unclear. Notably, *n*-1-dodecene was present in moderate abundance and in its hydrated form, i.e. *n*-dodecanol, is a significant constituent of *C. rotundus* essential oil component of the tuber/rhizome [3], and so its presence and relative abundance is consistent with the observed terpenoids characteristic of *C. rotundus*.

The two branched alkanes 5,5-diethyltridecane and 5,5-diethylpentadecane were detected in the TD-GC-MS and these ethyl- branched alkanes of C_{17} and C_{19} are typical of microorganisms, i.e. bacteria, algae and fungi [4], [5], [6], [7], [8]. Given their association with the terpenoids (see main text) it again suggests that they derive from a microorganism associated with *C. rotundus* tubers and rhizomes (see main text). An alkyl cyclohexane with a C_9 saturated side chain was also detected; odd carbon numbered alkyl cyclohexanes can be indicative of an algal source (their even numbered analogues are indicative of bacteria [9]), which would be consistent with

the lakes and swamp margins environment of *C. rotundus*, though the precise origin here is uncertain.

The pyrolysate TIC identified components indicative of 'black carbon', i.e. charcoal or soot [11], [12], [13], [14]. Benzene was the major compound present in the pyrolysate (~45% of total quantified pyrogram peak area), along with significant amounts of toluene (~16%) and minor quantities of xylenes (~3% total). More notable was significant amounts of naphthalene, with a lesser amount of biphenyl, also typical of chars [11], [12], [13], [14]. The protein marker benzonitrile [2] was present in significant abundance (~17%) fairly typical of previously characterised protein-containing chars [11], [12], [13], [14], although its abundance here could be indicative of a higher protein diet compared to other individuals at Al Khiday. The unsaturated compounds 1-butene and 1-pentene were present in minor abundance in the char, although their origin in this context is uncertain. Furan [2] was very tentatively identified in the pyrogram and in the absence of other carbohydrate markers [2] may be indicative of a degraded carbohydrate component, which would be consistent with the presence of the starch-rich *C. rotundus* (~24% starch [17]. The presence of the main combustion markers fluoranthene and pyrene, along with phenanthrene, albeit as trace components, supports the evidence for cooking/expose to fire in this sample and is consistent with the 'char' markers observed in the pyrogram.

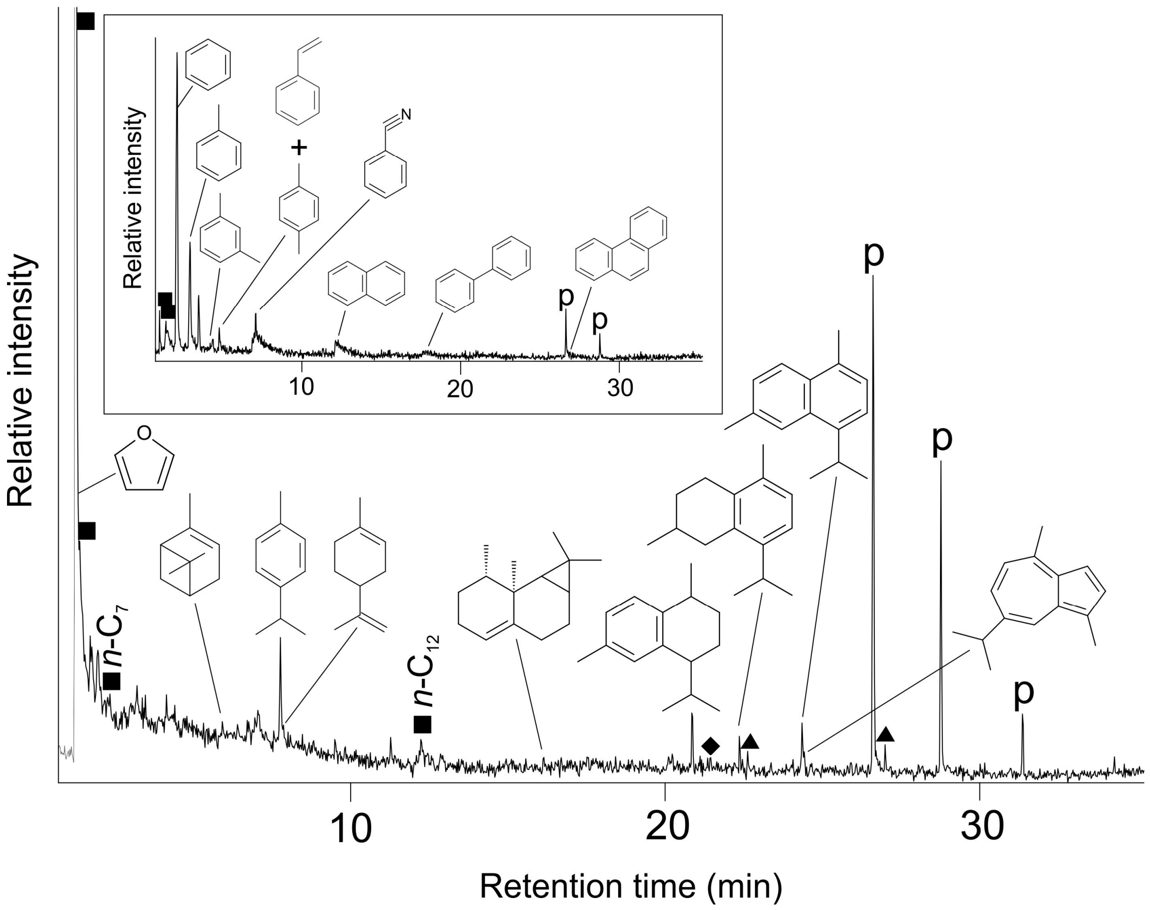

Figure S3 Reconstructed total ion chromatogram of the thermal desorption profile (310ºC for 10s) of human calculus, Burial 10-I, 0.92mg. Peak identities (x indicates carbon chain length): filled squares, Cx indicates alkenes; filled triangles indicates C_{17} and C_{19} 5,5-diethyl branched alkanes; filled diamond, Cx indicates alkylcyclohexane. Also shown are the structures of furan, three monoterpenoid compounds identified: α-pinene, *p*-cymene and limonene, and five sesquiterpenoid compounds identified: calarene (β-gurjunene), calamenene, calamene, cadalene and guaiazulene. Inset displays a reconstructed total ion chromatogram of the pyrolysis profile (610ºC for 10s) of this sample, after thermal desorption (310ºC for 10s). Peak identities: filled squares, Cx indicates alkenes. Also shown are the structures of nine aromatic compounds identified: benzene, toluene, m-xylene, styrene, *p*-xylene (coeluting with styrene), benzonitrile, naphthalene, biphenyl and the polynuclear aromatic hydrocarbon, phenanthrene. (p = pyroprobe contaminants)

Burial 93 Neolithic (calculus: 1.44mg)

The thermal desorption total ion chromatogram (TIC) revealed very few detectable components, indicating the virtual absence of any free, thermally extractable organic components in this

sample. It should be noted, however, that highly polar material could have been present, which would not have successfully eluted from the column, or indeed volatilised sufficiently in the probe. The components detected were 1,3-pentadiene, chloromethane, acetone, hexane, a dimethyl-2-pentene and xylenes. Due to the trace quantities, no meaningful interpretation can be assigned to these constituents.

The pyrolysate TIC contained very little material. Components identified included 1-butene, 1-pentene, penta-1,3-diene, penta-1,4-diene, cyclohexa-1,3-diene, although their origin in this context is uncertain. Also observed was the carbohydrate pyrolysis marker acetone, although in isolation and in such low abundance its significance is also difficult to determine. Although very few compounds were observed, the presence and relative abundance of benzene (44% of total quantified pyrogram peak area), toluene (23%), ethyl benzene and xylenes (4%), benzonitrile (2%), naphthalene and biphenyl, suggest a 'char', i.e. charcoal or soot, component in this sample. No other compounds were detected in the pyrogram.

Burial 96 Neolithic (calculus: 3.43mg)

The thermal desorption total ion chromatogram (TIC) revealed no detectable components, with the exception of chloromethane, indicating the essential absence of any free, thermally extractable lipids in this sample. It should be noted, however, that highly polar material could have been present, which would not have successfully eluted from the column, or indeed volatilised sufficiently in the probe.

The pyrolysate TIC contained little material. Components identified included 1-butene and penta-1,3-diene, although their origin in this context is uncertain. Although very few compounds were observed, the presence and relative abundance of benzene (56% of total quantified pyrogram peak area), toluene (16%), ethyl benzene and xylenes (2%), benzonitrile (10%), naphthalene and biphenyl, suggest a 'char', i.e. charcoal or soot, component in this sample. No other compounds were detected in the pyrogram.

Burial 103 Neolithic (calculus: 2.01mg)

The thermal desorption total ion chromatogram (TIC) (Fig. 1) revealed a significant number of monoterpenoids (C_{10}) and sesquiterpenoids (C_{15}), albeit in minor to moderate abundance. Three monoterpenoids were identified, i.e. α-pinene (minor trace), *p*-cymene and limonene (trace) (for

the significance of this latter monoterpenoid, see Meroitic sample 74 and discussion). The sesquiterpenoids observed were, in order of retention time (volatility), calarene (β-gurjunene), two unidentified sesquiterpenoids, calamenene, calamene and cadalene. Again, these terpenoids have previously all been found in the tubers and rhizomes of purple nut sedge, *C. rotundus* and their unusual natural occurrence combined with their relative abundance to each other point to the same plant source identified in pre-Mesolithic sample 35 and Neolithic sample 10-I (see Table 2 and main text).

The unsaturated compounds 1-butene, 1-pentene, 1,3-pentadiene, a methyl pentene and an octene were present in moderate abundance, possibly deriving from lipid or proteinaceous material in the sample [2], and perhaps produced at this relatively low temperature due to matrix effects. The additional presence of benzene and toluene may also derive from a proteinaceous component in the dental calculus, although also see 'pyrolysate' (below). A methyl cyclohexanone was present in moderate abundance although its origin here is uncertain. Notably, *n*-1-dodecene and n-dodecane were present in moderate abundance and the hydrated form of the former, i.e. *n*-dodecanol, is a significant constituent of *C. rotundus* essential oil component of the tuber/rhizome [3], and so its presence and relative abundance is consistent with the observed terpenoids characteristic of *C. rotundus*.

The branched alkane 5,5-diethyltridecane was detected in the TD-GC-MS and this C_{17} ethyl-branched alkane is typical of microorganisms, i.e. bacteria, algae and fungi [4], [5], [6], [7], [8]. Given its association with the terpenoids (see main text) it again suggests that it derives from a microorganism associated with *C. rotundus* tubers and rhizomes. A number of alkyl cyclohexanes with C_9, C_{11}, and C_{13} saturated side chains were also detected; odd carbon numbered alkyl cyclohexanes can be indicative of an algal source (their even numbered analogues are indicative of bacteria [9]), which would be consistent with the lakes and swamp margins environment of *C. rotundus*, though the precise origin here is uncertain. The chlorinated compounds, chlorobenzene and 2-chlorotoluene, were also detected in moderate to minor abundance. These chlorinated constituents are known to occur in microorganisms exposed to a saline environment (see main text). Sulphur dioxide was detected, and its generation at ~300°C suggests an organo-sulphate origin, possibly lignosulphonates formed from the interaction of lignin in the woody parts of plants with sulphates often present (e.g. gypsum) in significant abundance in saline lakes [18], [19], [20]. Sulphur dioxide is known to be a major pyrolysis product of lignosulphonates (see main text), and its organic nature would facilitate sulphur

dioxide generation at this relatively low temperature, although possible inference this can be no more than speculative and further research is necessary.

The pyrolysate TIC identified components indicative of 'black carbon', i.e. charcoal or soot [11], [12], [13], [14]. Benzene was the major compound present in the pyrolysate (~51% of total quantified pyrogram peak area), along with significant amounts of toluene (~10%) and minor quantities of ethyl benzene and xylenes (~2% total). More notable was significant amounts of naphthalene, with a lesser amount of biphenyl and methyl biphenyl, also typical of chars [11], [12], [13], [14]. The protein marker benzonitrile [2] was present in significant abundance (~16%) fairly typical of previously characterised protein-containing chars [11], [12], [13], [14], although this and the presence of pyridine (1% of total quantified pyrogram peak area), another protein marker [2], it could be indicative of a relatively higher protein diet compared to other individuals at Al Khiday. The unsaturated compounds 1-butene, penta-1,3-diene and penta-1,4-diene were present in minor abundance in the char, although their origin in this context is uncertain. No carbohydrate markers indicative of starch [15] could be detected, which may suggest diagenetic changes given the resilient biomarkers characteristic of *C. rotundus* present in the TD-GC-MS and the known susceptibility of carbohydrates to degradation (carbohydrates>DNA>proteins>lipids). The presence of the main combustion marker pyrene, along with phenanthrene, albeit as trace components, supports the evidence for cooking/expose to fire in this sample and is consistent with the 'char' markers observed in the pyrogram.

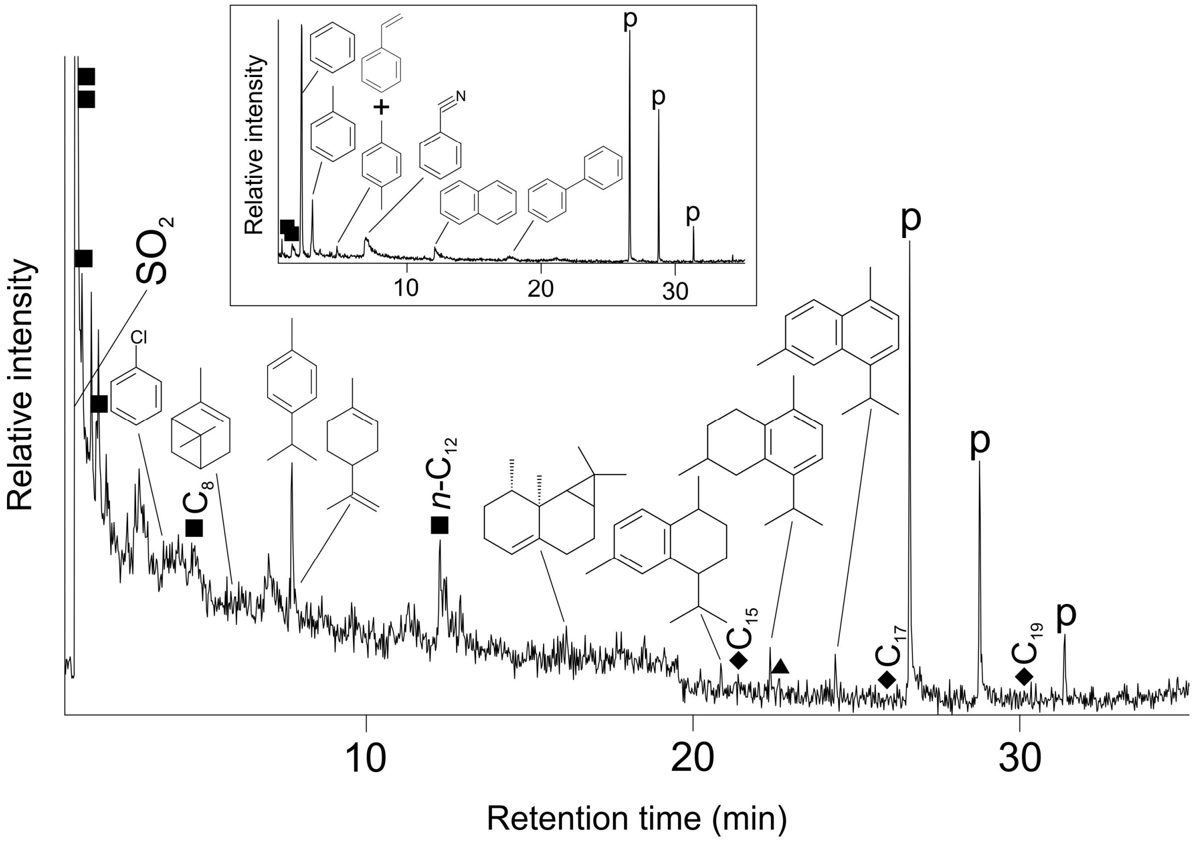

Figure S4 Reconstructed total ion chromatogram of the thermal desorption profile (310ºC for 10s) of human calculus, Burial 103, 2.01mg. Peak identities (x indicates carbon chain length): filled squares, Cx indicates alkenes; filled triangle indicates C_{17} 5,5-diethyl branched alkane; filled diamond, Cx indicates alkylcyclohexanes. Also shown are the structures of chlorobenzene, three monoterpenoid compounds identified: α-pinene, *p*-cymene and limonene, and four sesquiterpenoid compounds identified: calarene (β-gurjunene), calamenene, calamene and cadalene. SO_2 indicates sulphur dioxide. Inset displays a reconstructed total ion chromatogram of the pyrolysis profile (610ºC for 10s) of this sample, after thermal desorption (310ºC for 10s). Peak identities: filled squares, Cx indicates alkenes. Also shown are the structures of seven aromatic compounds identified: benzene, toluene, styrene, *p*-xylene (coeluting with styrene), benzonitrile, naphthalene and biphenyl. (p = pyroprobe contaminants)

Burial 47 Meroitic (calculus: 2.62mg)

The separate step of thermal desorption was not carried out on this sample, so it underwent pyrolysis only at 610°C. The TIC identified a significant number of organic components. Of those identified, benzene (~7% of total quantified pyrogram peak area), toluene (~23%), ethyl benzene and o-, m- and p-xylenes (6%) are likely to be indicative of 'black carbon', i.e. charcoal

or soot [11], [12], [13], [14], despite the relatively low abundance of benzene compared to toluene in this sample. Also notable were naphthalene and biphenyl, also typical of chars [11], [12], [13], [14], albeit in relatively minor abundance. The protein marker benzonitrile [2] was present in minor abundance (~0.5%) and although low for protein-containing chars [11], [12], [13], [14], the presence of a number of addition protein markers [2], i.e. pyrazole, 4-methylpyridine, styrene and indene (collectively, ~6% of total quantified pyrogram peak area), is indicative of a moderate protein component in the diet. Also notable is the carbohydrate marker, acetone (~12% of total quantified pyrogram peak area)), and indicative of a significant degraded carbohydrate component [2].

The pyrogram revealed a series of *n*-1-alkenes and *n*-alkanes, carbon numbers C_{10} to C_{15}, present in moderate abundance, and typical of fat/oil derived acyl lipids. In addition to these were a series of methyl branched alkanes, carbon numbers C_{12} to C_{17}, which is indicative of a bacterial lipid input in this sample. Also notable were the C_4 to C_9 *n*-1-alkenes, with no *n*-alkane analogue, and the polyunsaturated hydrocarbons buta-1,3-diene, penta-1,3-diene, hexa-1,3-diene, a heptadiene, a nonadiene, cyclopenta-1,3-diene, cyclohexa-1,3-diene, cyclohexa-1,4-diene, a methyl cyclohexadiene, a methyl cycloheptadiene, both present in significantly greater abundance than the C_{10} to C_{15}, alkene/alkane doublets, possibly suggesting a significant polyunsaturated lipid component in this sample.

Burial 74 Meroitic (calculus: 5.46mg)

The thermal desorption total ion chromatogram (TIC) (Fig. 3 main text) is dominated by a very significant number of monoterpenoids (C_{10}) and sesquiterpenoids (C_{15}) in minor to major abundance. A number of monoterpenoids were identified, i.e. α-pinene, trans-carene and *p*-cymene, limonene, β-phelladrene, 2-carene and *p*-cymenene. The TD chromatogram revealed a very complex suite of sesquiterpenoids which included, in order of retention time (volatility), calarene (gurjunene), norrotundene, rotundene, γ-muurolene, α-copaene, cubinene, α-cedrene, an unidentified, cypera-2,10-diene, γ-selinene, α-muurolene, γ-cadinene(?), calamenene (major component), calacorenes (x3), α-cadinene, calamene (major component), a dehydrocadalene, cadalene (major component), an isomer of cadalene and guaiazulene. These terpenoids have previously been found in the tubers and rhizomes of purple nut sedge, *Cyperus rotundus*, and their unusual natural occurrence and their collective presence in the dental calculus very strongly suggests this specific plant source (see Table 2 and main text). Notably, the *n*-1-dodecene is a

major component, with no *n*-dodecane analogue, and in its hydrated form, i.e. *n*-dodecanol, is a significant constituent of *C. rotundus* essential oil component of the tuber/rhizome [3], and so both its presence and its relative abundance compared to the observed terpenoids characteristic of *C. rotundus*, is strongly indicative of the presence of this plant. The absence of series of *n*-1-alkenes and *n*-alkanes in this sample, which might otherwise suggest a lipid origin for some or all of these components, further supports this interpretation.

A suite of branched alkanes constituted a minor to moderate component of the free compounds identified in the TD-GC-MS. These include 5,5-diethyltridecane, 5,5-diethyltetradecane (minor), 5,5-diethylpentadecane, 5,5-diethylheptadecane, 5,5-diethylnonadecane, 3,3-diethyltridecane, 3,3-diethylpentadecane, 3,3-diethylheptadecane, 3-ethyl-3-methyltridecane, 3-ethyl-3-methylpentadecane, 3-ethyl-3-methylheptadecane, 3-ethyl-3-methylnonadecane, 5-butyl-5-ethylpentadecane, 5,5-dibutyldecane, 5,5-dibutyldodecane, 5,5-dibutyltetradecane, and 6,6-diethyloctadecane. These methyl, ethyl and butyl- branched alkanes of C_{16} to C_{23} are typical of microorganisms, i.e. bacteria, algae and fungi [4], [5], [6], [7], [8]. Given their association with the terpenoids (see main text) it suggests that they derive from a microorganism associated with *C. rotundus* tubers and rhizomes. A series of alkyl cyclohexanes with C_9, C_{11}, C_{13}, C_{15} and C_{17} saturated side chains were also detected in minor to moderate abundance; odd carbon numbered alkyl cyclohexanes can be indicative of an algal source (their even numbered analogues are indicative of bacteria [9]), which would be consistent with the lakes and swamp margins environment of *C. rotundus*, though the precise origin here is uncertain.

A dimethyl-2-pentene, benzaldehyde and α-ionene (1,2,3,4-tetrahydro-1,1,6-trimethylnaphthalene) were observed in moderate abundance; these three compounds (and their isomers) are known to occur in *Prunus* species [21], [22] , although other evidence would be needed in order to even tentatively understand the possible significance of these compounds here. The two alkenes 2-octene and 1-tridecene, a methyl cyclopentane, a methyl cyclohexene and benzene were also identified, although in the absence of other related compounds their origin is unclear. Although only present as a very minor component, the polynuclear aromatic hydrocarbon pyrene was also detected. The chlorinated hydrocarbon chlorobenzene was detected as a minor but notable component; chlorinated compounds are known to occur in microorganisms exposed to a saline environment (see mean text) and the largely aromatic nature of the constituents identified in this sample of dental calculus may explain the dominance of the aromatic chloro- compound. Sulphur dioxide was detected as a major component, and its

generation at ~300°C suggests an organo-sulphate origin, possibly lignosulphonates formed from the interaction of lignin in the woody parts of plants with sulphates often present (e.g. gypsum) in significant abundance in saline lakes [18], [19], [20]. Sulphur dioxide is known to be a major pyrolysis product of lignosulphonates (main text), and its organic nature would facilitate sulphur dioxide generation at this relatively low temperature, although possible inference this can be no more than speculative and further research is necessary.

The pyrolysate TIC identified components indicative of 'black carbon', i.e. charcoal or soot [11], [12], [13], [14]. Benzene was the major compound present in the pyrolysate (~26% of total quantified pyrogram peak area), along with significant amounts of toluene (~12%) and minor quantities of ethyl benzene and o-, m- and p-xylenes (~3% total). Also notable was a significant amount of naphthalene, with lesser amounts of methyl naphthalene, biphenyl and methyl biphenyl, also typical of chars [11], [12], [13], [14]. The protein marker benzonitrile [2] was present in significant abundance (~23%) fairly typical of previously characterised protein-containing chars [11], [12], [13], [14], although this and the presence of moderate to major quantities of propanenitrile, butanenitrile, pyrazine, pyridine, 2-methylpyridine, 4-methylpyridine, 2-chloropyridine, 3?-methylbenzonitrile 2,3-pyridine carbonitrile and phenylpyridine, also protein pyrolysis markers [2], is indicative of a higher protein diet compared to most other individuals at Al Khiday.

The unsaturated compounds 1-butene, 1,3-butadiene, and 1,3-pentadiene were present in significant abundance in the char, although their origin in this context is uncertain. No carbohydrate markers indicative of starch [15] could be detected, which may suggest diagenetic changes given the resilient biomarkers characteristic of *C. rotundus* present in the TD-GC-MS and the known susceptibility of carbohydrates to degradation (carbohydrates>DNA>proteins>lipids). The presence of the combustion markers 9H-fluoren-9-one, phenathrene, fluoranthene and pyrene supports the evidence for cooking/expose to fire in this sample and is consistent with the 'char' markers observed in the pyrogram. The chlorinated compounds chlorobenzene and 2-chloropyridine, were also detected in moderate to minor abundance in the pyrogram. Again, chlorinated constituents are known to occur in microorganisms exposed to a saline environment (see main text), and the aromatic nature of the constituents identified may explain the dominance of the aromatic chloro- compounds here. Sulphur dioxide observed in the TD was also present in the pyrolysate, albeit in a lower

abundance, which may be due to matrix effects in the dental calculus samples. Its likely origin is the same as that for the gas generated in the TD.

Burial 106 Meroitic (calculus: 4.98mg)

The thermal desorption total ion chromatogram (TIC) revealed very few detectable components, indicating the virtual absence of any free, thermally extractable organic components in this sample. It should be noted, however, that highly polar material could have been present, which would not have successfully eluted from the column, or indeed volatilised sufficiently in the probe. Of the trace components which were present chloromethane was the major constituent, with benzene and toluene also detected. Chlorinated compounds are known to occur in microorganisms exposed to a saline environment (see main text), which may explain the presence of the chloromethane, but for the ubiquitous aromatic compounds, benzene and toluene, the trace quantities mean no meaningful interpretation can be assigned to these constituents.

The pyrolysate TIC identified components indicative of 'black carbon', i.e. charcoal or soot [11], [12], [13], [14]. Benzene was the major compound present in the pyrolysate (~27% of total quantified pyrogram peak area), along with significant amounts of toluene (~11%) and moderate quantities of ethyl benzene and xylenes (~8% total). More notable were significant amounts of naphthalene, methyl naphthalene, biphenyl and methyl biphenyl, also typical of chars [11], [12], [13], [14]. The protein marker benzonitrile [2] was present in significant abundance (~13%) fairly typical of previously characterised protein-containing chars [11], [12], [13], [14], although this and the presence of two other protein markers [2], pyridine (~3% of total quantified pyrogram peak area) and indene (~1%), could be indicative of a relatively higher protein diet compared to other individuals at Al Khiday. The unsaturated compounds 1-butene, 1-pentene, 1,3-pentadiene, 1,3-hexadiene and 1,4-hexadiene were present in significant abundance in the char, although their origin in this context is uncertain . Also notable is acetone (~2%), which is a pyrolysis carbohydrate marker [2] and possibly indicative of a carbohydrate-starch component. The presence of the main combustion markers fluoranthene and pyrene, along with phenanthrene, albeit as very minor components, supports the evidence for cooking/expose to fire in this sample and is consistent with the 'char' markers observed in the pyrogram.

Although it can only be highly tentative, *p*-cymene seen in samples 41, 64 (along with dodecene, possibly deriving from dodecanol; see main text), might be indicative of *C. rotundus*. The

absence of other terpenoids may be due to the expected greater microbial and diagenetic resistance of this aromatic monoterpenoid.

‡ Absent due to prevailing humid/wet environment at this time. The greater relative abundance of limonene present in the Meroitic sample (6% of volatile thermal extract) compared to the Neolithic samples (1-2% of volatile thermal extract) correlates with previous studies on terpenes in plants where the abundance of this monoterpene increases with a decrease in humidity and precipitation [23]; this reflection of the environment becoming increasingly arid from pre-Mesolithic to Meroitic corresponds with the previous stable isotope and palaeoenvironmental climate studies [24], [25], [26], [27], [28].

References

1. Buckley SA, Stott AW, Evershed RP (1999) Studies of organic residues from ancient Egyptian mummies using high temperature-gas chromatography-mass spectrometry and sequential thermal desorption-gas chromatography-mass spectrometry and pyrolysis gas chromatography-mass spectrometry. Analyst 124:443–452.

2. Simmonds PG (1970) Whole microorganisms studied by pyrolysis-gas chromatography-mass spectrometry: significance for extraterrestrial life detection experiments. Appl Microbiol 20(4): 567-572.

3. Ghannadi A, Rabbani M, Ghaemmaghami L, Malekian N (2012) Phytochemical screening and essential oil analysis of one of the Persian sedges; *Cyperus rotundus* L. International Journal of Pharmaceutical Sciences and Research 3(2): 424-427.

4. Greenwood PF, Arouri KR, Logan GA, Summons RE (2004) Abundance and geochemical significance of C_{2n} dialkylalkanes and highly branched C_{3n} alkanes in diverse Meso- and Neoproterozoic sediments. Org Geochem 35: 331-346. doi: 10.1016/j.orggeochem.2003.10.013

5. Kenig F, Simons DJH, Anderson KB (2001) Distribution and origin of ethyl-branched alkanes in a Cenomanian transgressive shale of the Western Interior Seaway (USA). Org Geochem 32: 949-954. doi: 10.1016/S0146-6380(01)00049-3

6. Kenig F, Simons DJH, Crich D, Cowen JP, Ventura GT, et al. (2003) Branched aliphatic alkanes with quaternary substituted carbon atoms in modern and ancient geologic samples. Proc Natl Acad Sci USA 100: 12554-12558. doi: 10.1073/pnas.1735581100

7. Kenig F, Simons DJH, Crich D, Cowen JP, Ventura GT, et al. (2005) Structure and distribution of branched aliphatic alkanes with quaternary carbon atoms in Cenomanian and Turonian black shales of Pasquia Hills (Saskatchewan, Canada). Org Geochem 36: 117-138. doi: 10.1016/j.orggeochem.2004.06.014

8. Hautville Y, Michels R, Malartre F, Elie M, Trouiller A (2007) Tracing of variabilities within a geological barrier by molecular organic geochemistry. Case of the Callovo-Oxfordian sedimentary series in the East of the Paris Basin (France). Appl Geochem 22: 736-759. doi: 10.1016/j.apgeochem.2006.10.003

9. Hoffmann CF, Foster CB, Powell TG, Summons RE (1987) Hydrocarbon biomarkers from Ordovician sediments and the fossil alga *Gloeocapsomorpha prisca* Zalessky 1917. Geochim Cosmochim Acta 51: 2681-2697. doi: 10.1016/0016-7037(87)90149-9

10. Hiatt MH (1983) Determination of volatile organic compounds in fish samples by vacuum distillation and fused silica capillary gas chromatography/mass spectrometry. Anal Chem 55: 506-516. doi: 10.1021/ac00254a022

11. Kaal J, Martínez Cortizas A, Eckmeier E, Costa Casais M, Santos Estévez M, et al. (2008) Holocene fire history of black colluvial soils revealed by pyrolysis-GC/MS: a case study from Campo Lameiro (NW Spain). J Archaeol Sci 35: 2133-2143. doi: 10.1016/j.jas.2008.01.013

12. Kaal J, Brodowski S, Baldock JA, Nierop KGJ, Martínez Cortizas A (2008) Characterisation of aged black carbon pyrolysis-GC/MS, thermally assisted hydrolysis and methylation (THM), direct and cross polarisation ^{13}C nuclear magnetic resonance (DP/CP NMR) and the benzenepolycarboxylic acid (BPCA) method. Organic Geochemistry 39: 1415-1426. doi: 10.1016/j.orggeochem.2008.06.011

13. Kaal J, Martínez-Cortizas A, Buurman P, Criado Boado F (2008) 8000 yr of black carbon accumulation in a colluvial soil from NW Spain. Quaternary Research 69: 56-61. doi: 10.1016/j.yqres.2007.10.005

14. Kaal J, Martínez Cortizas A, Nierop KGJ (2009) Characterisation of aged charcoal using a coil probe pyrolysis-GC/MS method optimised for black carbon. J Anal Appl Pyrolysis 85: 408-416. doi: 10.1016/j.jaap.2008.11.007

15. McCobb LME, Briggs DEG, Evershed RP, Hall AR, Hall RA (2001) Preservation of fossil seeds from a 10th century AD cess pit at Coppergate, York. J Archaeol Sci 28: 929-940. doi: 10.1006/jasc.2000.0617

16. Peng Y, Wu S (2011) Fast pyrolysis characteristics of sugarcane bagasse hemicellulose. Cellulose Chemistry and Technology 45(9-10): 605-612.

17. Umerie SC, Ezeuzo HO (2000) Physicochemical characterization and utilization of *Cyperus rotundus* starch. Bioresour Technol 72: 193-196. http://dx.doi.org/10.1016/S0960-8524(99)00103-0

18. Salama RB (1987) The evolution of the River Nile. The buried saline rift lakes in Sudan – I. Bahr El Arab Rift, the Sudd buried saline lake. Journal of African Earth Sciences 6: 899-913. doi: 10.1016/0899-5362(87)90049-2

19. Salama RB (1994) The Sudanese buried saline lakes. Geological Society of America Special Papers 289: 33-47.

20. Farah EA (1999) Groundwater quality and hydrogeologic conditions in the Khartoum area, Sudan. Journal of Geosciences. Osaka City University 2(3): 45-53.

21. Gómez E, Ledbetter CA, Hartsell PL (1993) Volatile compounds in apricot, plum, and their interspecific hybrids. J Agric Food Chem 41: 1669-1676. doi: 10.1021/jf00034a029

22. Pino JA, Quijano CE (2012) Study of the volatile compounds from plum (*Prunus domestica* L. cv. Horvin) and estimation of their contribution to the fruit aroma. Ciência e Tecnologia de Alimentos 32: 76-83. doi.org/10.1590/S0101-20612012005000006

23. Lago JHG, Fávero OA, Romoff, P (2006) Microclimatic factors and phenology influences in the chemical composition of the essential oils from *Pittosporum undulatum* Vent. leaves. J Braz Chem Soc 17: 1334-1338. doi.org/10.1590/S0103-50532006000700021

24. Usai D, Salvatori S, Faroug MA, Di Matteo A, Iacumin P, et al. (in press) Archaeological evidence at Al Khiday. New insight on the prehistory and history of Central Sudan. In: Anderson

JR, Welsby DA, editors. The Fourth Cataract and Beyond. Proceedings of the 12th International Conference for Nubian Studies, the British Museum, London, 1st – 6th August, 2010. Louvain: Peeters.

25. Usai D, Salvatori S, Iacumin P, Di Matteo A, Jakob T, et al. (2010) Excavating a unique pre-Mesolithic cemetery in Central Sudan. Antiquity 84(323). Available: http://www.antiquity.ac.uk/projgall/usai323/. Accessed 29 December 2013

26. Williams MAJ, Adamson DA (1973) The physiography of the Central Sudan. Geogr J 139: 498-508. doi: 10.2307/1795032

27. Williams MAJ (2009) Late Pleistocene and Holocene environments in the Nile basin. Global Planet Change 69: 1-15. doi: 10.1016/j.gloplacha.2009.07.005

28. Lario J, Sanchez-Moral S, Fernandez V, Jimeno A, Menendez M (1997) Palaeoenvironmental evolution of the Blue Nile (Central Sudan) during the Early and Mid-Holocene (Mesolithic-Neolithic Transition). Quat Sci Rev 16: 583-588. doi: 10.1016/S0277-3791(96)00053-4
