# Supplementary material for: Dental Calculus Reveals Unique Insights into Food Items, Cooking and Plant Processing in Prehistoric Central Sudan
Source: PLoS One. 2014 Jul 16;9(7):e100808. doi: 10.1371/journal.pone.0100808 (PMC4100759; doi:10.1371/journal.pone.0100808)
Supplement: Information S2 — Starch granule morphology and general distribution of types. (PDF) [file pone.0100808.s002.pdf]

S2. Starch granule morphology and general distribution of types.

Starch type	General Morphology	Hilum	Extinction cross	Visible diagenetic effects	Other	Occurrence
***Type 1*: polyhedral**	Polyhedral, to sub-polyhedral, 18-25µm. Lamellae absent. It has similarities with *Cyperus rotundus* L.	Clear, central hilum, small fissures from the centre give it an almost stellate aspect. 'Stellate' hilum is absent in modern *C. rotundus* L.	Distinct, centric, with high degree of polarisation in most cases. It forms 4 arms and extends the length of the granule, slightly enlarging toward granule edges. Similar to modern *C. rotundus L.*	None	Some granules still lodged within remains of the cellular wall. Some granules still partially embedded in the calculus matrix	Pre-Mesolithic and Meroitic
***Type 2*: large, oval**	Oval to sub-triangular, between 20-35 µm, suggesting a degree of diversity**.** Numerous thin lamellae	Evident under polarized light as a fissure is visible in the central part of the granules	Distinct, centric to eccentric, appears as darker area in the central part of the granule with 4 or more asymmetric arms diverging from the central area. Arms have different width, possibly due to alteration during cooking	Diagenetic effects not present in the Pre-Mesolithic samples, but clearly visible in the Neolithic and Meroitic	Some starch granules occurred in groups of two or three, still lodged within remains of the cellular wall. The granules have similarities with those of the Triticeae Tribe, of the Family Poaceae	Few in pre-Mesolithic, more common in the Neolithic
***Type 3*: smaller, round**	Small, round, 5-15µm	Not visible	Similar to type 2	Diagenetic effect has altered the hilum in the Neolithic samples	Likely to be smaller granule of type 2, as one very similar was seen lodged with *type 2,* similar to bimodal starches of the Triticeae tribe	Neolithic and Meroitic
