# Supplementary figures and images for: Dental Calculus Reveals Unique Insights into Food Items, Cooking and Plant Processing in Prehistoric Central Sudan
Source: PLoS One. 2014 Jul 16;9(7):e100808. doi: 10.1371/journal.pone.0100808 (PMC4100759; doi:10.1371/journal.pone.0100808)

## Slide 1
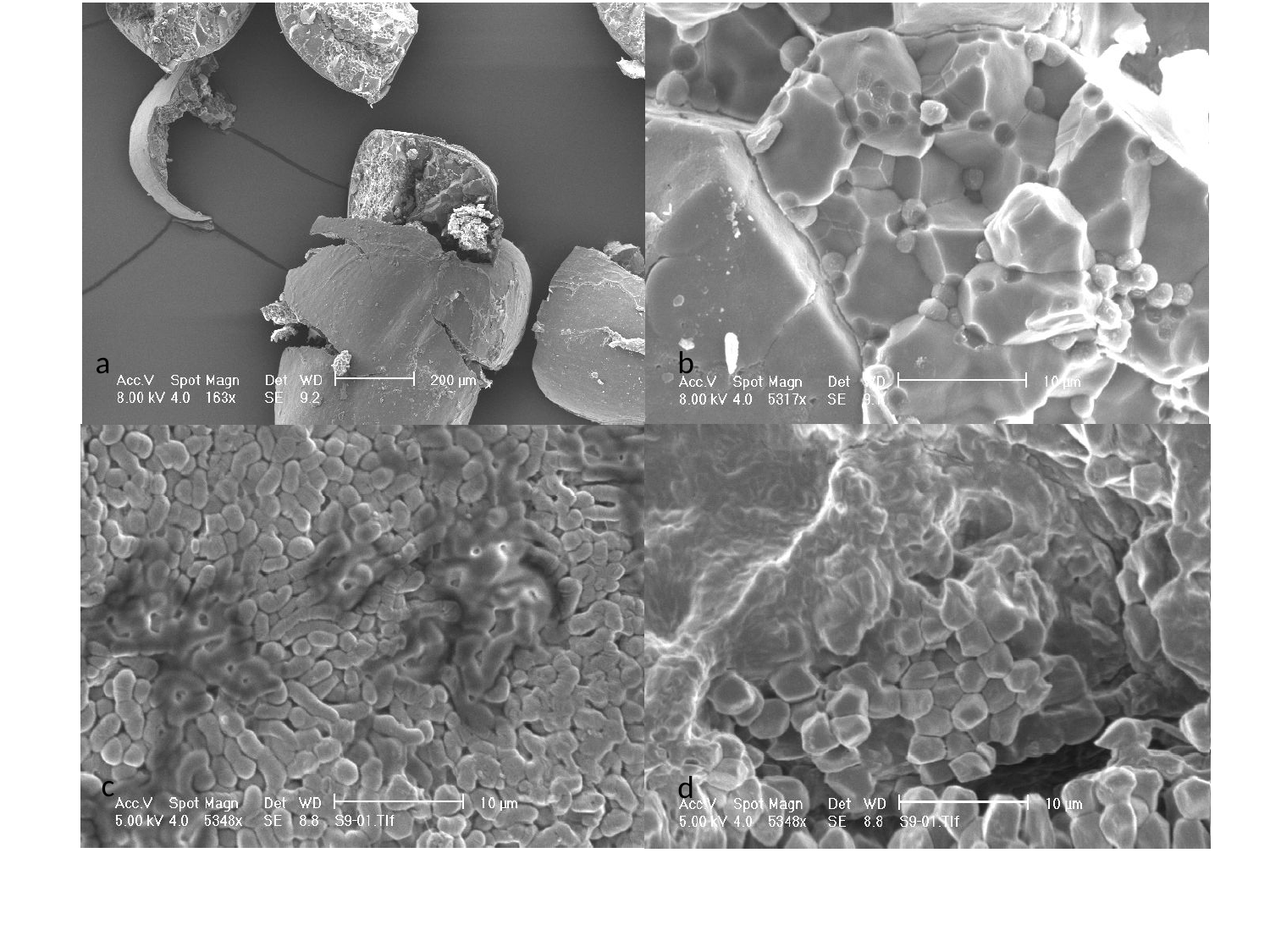

a
b
c
d

Supplement: Information S3 — Variable gelatinization of starch granules following open fire cooking. (a) raw seed (163 magnifications); (b) Internal structure of raw seed, (5317 magnifications); (c) partially gelatinized starch granules (5348 magnifications); (d) raw to fully gelatinized starch granules (5348 magnifications). (PPTX) [file pone.0100808.s003.pptx]
